# Cytosolic delivery of siRNA by ultra-high affinity dsRNA binding proteins

**DOI:** 10.1093/nar/gkx546

**Published:** 2017-06-21

**Authors:** Nicole J. Yang, Monique J. Kauke, Fangdi Sun, Lucy F. Yang, Katie F. Maass, Michael W. Traxlmayr, Yao Yu, Yingda Xu, Robert S. Langer, Daniel G. Anderson, K. Dane Wittrup

**Affiliations:** 1 Department of Chemical Engineering, Massachusetts Institute of Technology, Cambridge, MA 02139, USA; 2 Department of Biological Engineering, Massachusetts Institute of Technology, Cambridge, MA 02139, USA; 3 David H. Koch Institute for Integrative Cancer Research, Massachusetts Institute of Technology, Cambridge, MA 02139, USA; 4 Protein Analytics, Adimab LLC, Lebanon, NH 03766, USA; 5 Harvard-MIT Division of Health Science and Technology, Massachusetts Institute of Technology, Cambridge, MA 02139, USA; 6 Institute for Medical Engineering and Science, Massachusetts Institute of Technology, Cambridge, MA 02139, USA

## Abstract

Protein-based methods of siRNA delivery are capable of uniquely specific targeting, but are limited by technical challenges such as low potency or poor biophysical properties. Here, we engineered a series of ultra-high affinity siRNA binders based on the viral protein p19 and developed them into siRNA carriers targeted to the epidermal growth factor receptor (EGFR). Combined *in trans* with a previously described endosome-disrupting agent composed of the pore-forming protein Perfringolysin O (PFO), potent silencing was achieved *in vitro* with no detectable cytotoxicity. Despite concerns that excessively strong siRNA binding could prevent the discharge of siRNA from its carrier, higher affinity continually led to stronger silencing. We found that this improvement was due to both increased uptake of siRNA into the cell and improved pharmacodynamics inside the cell. Mathematical modeling predicted the existence of an affinity optimum that maximizes silencing, after which siRNA sequestration decreases potency. Our study characterizing the affinity dependence of silencing suggests that siRNA-carrier affinity can significantly affect the intracellular fate of siRNA and may serve as a handle for improving the efficiency of delivery. The two-agent delivery system presented here possesses notable biophysical properties and potency, and provide a platform for the cytosolic delivery of nucleic acids.

## INTRODUCTION

Protein-based systems for delivering siRNA can potentially circumvent some of the challenges facing nanoparticle-based systems, such as accumulation in the liver (1,2). Although there exists multiple examples of protein-based delivery methods (3–7), they generally suffer from low potencies, complex preparation strategies, or poor pharmacokinetics and biodistribution properties stemming from high positive charge (8,9). Hence, multiple technical barriers still need to be overcome for protein-based methods to become a competitive delivery strategy.

Proteinaceous delivery vehicles commonly require both a carrier functionality, provided by a moiety that is chemically conjugated to or non-covalently complexed with siRNA, and an endosomal release functionality, which can be explicitly defined or embedded within the vehicle. For example, Dowdy *et al.* used the double-stranded RNA binding domain (dsRBD) of Protein Kinase R (PKR) fused with protein transduction domains (PTDs) to respectively bind and transport siRNA across the cell membrane (6,10).

Previously, we reported a multi-agent delivery system that also employed the dsRBD of PKR as a siRNA carrier. Administered together with an endosome-disrupting agent based on the pore-forming protein Perfringolysin O (PFO), and a receptor-clustering antibody that enhances siRNA uptake, efficient silencing was achieved *in vitro* (11). While the non-cationic and non-particulate nature of this delivery system suggested that desirable pharmacokinetics and biodistribution properties could be achieved *in vivo*, we observed that siRNA dissociated rapidly from the dsRBD during circulation, negating the targeting functionalities of the carrier protein.

To address this delivery limitation, we focused on developing siRNA carriers with higher affinities for siRNA. To this end, we chose the p19 protein of the *Carnatian Italian Ringspot Virus* (CIRV) as an alternative siRNA-binding scaffold. Like dsRBDs, p19 binds specifically to double-stranded RNA (dsRNA) independent of sequence, and not to single-stranded RNA (ssRNA) or DNA (12). However, in contrast to dsRBDs, p19 binds in a size-dependent manner to dsRNAs the length of siRNA, providing increased specificity (12,13). Importantly, p19 has a naturally higher affinity for siRNA (13,14), which provides an excellent backbone to further engineer ultra-high affinity siRNA carriers.

Although our prior work indicated that siRNA carriers with higher affinity would be required for successful *in vivo* targeting, strong binding can also come with potential drawbacks. Indeed, previous observations have supported the notion that excessively strong binding between siRNA and its delivery vehicle is undesirable, as it may prevent siRNA from being released and subsequently loaded onto the RNA-induced silencing complex (RISC) (15,16). For example, Schroeder *et al*. observed that stronger binding between siRNA and various PEI–lipid conjugates correlated with decreased silencing potency (15). Han *et al.* observed that continually increasing the polymer-siRNA binding strength eventually caused a decline in silencing potency (16). Despite such precedents, we reasoned that our delivery system is distinct from the previously reported formulations in that our siRNA carrier is physically separate from the endosome-disrupting agent. This modularity gave us the opportunity to isolate siRNA-carrier affinity as a true independent variable for characterizing its influence on silencing potency.

Here, we report the engineering of ultra-high affinity siRNA binding proteins based on the p19 scaffold and their development into targeted siRNA carriers. Combined with the PFO-based endosome-disrupting agent (17), potent silencing was achieved *in vitro* with no signs of cytotoxicity. Unexpectedly, higher carrier affinity continually increased the silencing potency even when there were no additional gains in siRNA uptake, suggesting that higher affinity also allowed for more efficient delivery to RISC downstream of siRNA internalization. Mathematical modeling predicted that this positive correlation between affinity and potency will eventually reverse, indicating the existence of an affinity optimum that maximizes silencing. In this study, a 20-fold improvement in potency was achieved by increasing affinity alone, suggesting that tuning siRNA affinity may provide an additional avenue for increasing the efficiency of delivery. Overall, our results imply that siRNA-carrier affinity may significantly influence the intracellular pharmacodynamics of siRNA, beyond its previously assigned roles in affecting particle stability and cellular uptake. Furthermore, the ultra-high affinity siRNA binders developed for this study may serve as useful tools for diverse applications involving dsRNA detection, isolation or sequestration.

## MATERIALS AND METHODS

### Cell lines

A431 cells (ATCC) and A431 cells stably transfected with destabilized EGFP (A431-d2EGFP) (11) were cultured in DMEM (ATCC) supplemented with 10% heat-inactivated FBS (Life Technologies). The A431-d2EGFP cells received 0.1 mg/mL G418 (Corning) in addition. All cell lines were maintained at 37°C and 5% CO_2_ in a humidified incubator.

### siRNA

Please refer to Supplementary Table S1 for sequences, modifications and vendor information.

### Affinity maturation of p19

Affinity maturation of p19 was performed using standard yeast surface display techniques as previously described (18) with select modifications. P19 was mutagenized by error-prone PCR and displayed on the surface of yeast fused to human Fc (IgG1). The resulting library was screened by fluorescence activated cell sorting (FACS) following kinetic sorting methods (19) where clones are selected for a slower dissociation rate. Three siRNAs were used for selection (Seq F, Seq I and Cy5-labeled AllStars negative control siRNA). Dissociation was performed in PBSA containing 55% mouse serum (EMD Millipore) at 37°C to mimic *in vivo* conditions. Unlabeled siRNA (unmodified Seq F) was added at a 100-fold molar excess over the estimated concentration of labeled siRNA. The final concentration of the competitor was between 1.7 and 2.5 μM. Six rounds of selections were performed in total, cycling between the different siRNAs twice to prevent specific binding to any one sequence. The competition time was increased from 15 minutes in the first selection cycle to 1 hour in the second cycle. Plasmids were isolated and sequenced from the enriched library as previously described (18).

### Protein expression and purification

The PFO-based endosome-disrupting agent (C225.2/PFO^T490A,L491V^) was prepared as previously described (17). The p19, p19-E6, p19-E18 clones and SUMO-E18 were expressed from the pE-SUMO vector (LifeSensors) in Rosetta 2 (DE3) *Escherichia coli* (Novagen) and purified by Talon metal affinity chromatography (Clontech) following previously described methods (17). Following cleavage of the SUMO tag, the p19 constructs were purified by anion exchange chromatography (AEX) and size-exclusion chromatography (SEC). AEX was performed using a HiTrap Q HP anion exchange column (GE Healthcare Life Sciences) with an increasing salt gradient (10–500 mM NaCl) in 20 mM Bis–Tris, pH 6.5. SEC was performed using a HiLoad 16/600 Superdex 75 pg column (GE Healthcare Life Sciences) in PBS. Analytical SEC was performed using a Superdex 75 10/300 GL column (GE Healthcare Life Sciences) or Superdex 200 Increase 10/300 GL column (GE Healthcare Life Sciences) in PBS. Detailed methods for the expression and purification of p19 are provided in Supplementary Methods.

### Dynamic light scattering

Targeted p19 constructs were analyzed at 5 μM (dimer concentration) in PBS (pH 7.4), either alone or complexed with siRNA (unmodified Seq F) at a molar ratio of 1:1 (p19 dimer: siRNA). For complexation, p19 and siRNA were incubated for 30 minutes at 4°C or on ice. Samples (50 μl each) were equilibrated to 25°C and analyzed with the DynaPro NanoStar Light Scatterer (Wyatt Technology) using the Dynamics software (Wyatt Technology). Each run consisted of 20 acquisitions (10 s per acquisition), and two runs were performed per sample.

### Biolayer interferometry (BLI)

All measurements were performed in citrate-phosphate buffer containing 100 mM NaCl, 0.1% BSA and 0.002% Tween-20 at 37°C using an Octet RED96 instrument (Pall ForteBio LLC). Biotinylated hFc-EGFR (prepared in-house (20)) or biotinylated siRNA (Seq 7) was captured on streptavidin-coated BLI tips (Pall ForteBio). P19-E6/siRNA and p19-E18/siRNA complexes were prepared by incubating the carriers and siRNA (unmodified Seq F) at a 1:1 molar ratio (p19 dimer: siRNA) for 30 min at 4°C immediately prior to BLI. Association was analyzed at various concentrations of the p19 constructs (2-fold dilutions from 10 to 0.16 nM for EGFR affinity measurements; 2-fold dilutions from 25 to 0.78 nM for siRNA affinity measurements with untargeted p19; 2-fold dilutions from 10 to 0.31 nM for siRNA affinity measurements with the untargeted p19 mutants). Dissociation was performed for between 30 min and 1 h. The buffer baseline from loaded tips was subtracted from the data, which were then globally fitted to a 1:1 binding model to obtain apparent affinities.

### MSD-SET

MSD-SET was performed as previously described (21) with minor modifications. Briefly, standard bind plates (Meso Scale Discovery) were coated with 100 nM of each p19 clone in PBS for 30 min at room temperature or overnight at 4°C, then blocked and washed. Samples were prepared by incubating 100 pM of biotinylated siRNA (Seq 7) with varying concentrations of each p19 clone (3-fold serial dilutions from 200 nM to 2 pM) in PBS, 0.1% BSA for 24 h at room temperature. Samples were then applied to plates coated with the respective p19 clone for 150 s with shaking to capture any free siRNA. Captured siRNA was detected with sulfotag-labeled streptavidin (Meso Scale Discovery) imaged on a MSD Sector Imager 2400 instrument (Meso Scale Discovery). The collected data was fitted to a quadratic equilibrium binding model (22) to obtain dissociation constants. Liquid handling was performed robotically to minimize variability.

### GFP silencing assays

A431 or A431-d2EGFP cells were seeded at a density of 15 000 cells/well in 96-well plates 16–20 h prior to the experiment. The p19 constructs were incubated with negative control siRNA (Qiagen) or GFP siRNA (GE Dharmacon) at a 1:1 molar ratio (p19 dimer: siRNA) for 30 min at 4°C or on ice. The p19/siRNA complexes were then serially diluted in DMEM containing 10% FBS and either 5 or 0.5 nM C225.2/PFO^T490A,L491V^. For competition experiments, incubation was performed with 20 nM p19-E18/siRNA complexes, 5 nM C225.2/PFO^T490A,L491V^ and varying concentrations (0–4 μM) of SUMO-E18 in complete media. Cells were transfected for 6 h, followed by incubation in fresh, complete media for an additional 18 h. Following trypsinization and neutralization (PBSA, 2% FBS), cells were analyzed on a BD LSR II HTS cytometer (BD Biosciences). Background from A431 cells was subtracted from all measurements, which were then normalized to that of untreated cells (when p19/siRNA or C225.2/PFO^T490A,L491V^ was used alone) or cells treated with the corresponding concentration of C225.2/PFO^T490A,L491V^ only (all other cases). Cell viability was measured immediately prior to analyzing GFP expression using the WST-1 reagent (Roche) as previously described (11). Background subtracted values were normalized to that of C225.2/PFO^T490A,L491V^-treated cells.

### PLK1 silencing assays

Transfection procedures for PLK1 were identical to those described above for GFP with the following exceptions. A431 cells were seeded at a density of 45 000 cells/well in 48-well plates for measuring knockdown of PLK1 mRNA; 18 000 cells/well in 96-well plates for measuring knockdown of PLK1 protein and 12 000 cells/well in 96-well plates for measuring cell viability. P19-E18 and p19^N15K,G16R^-E18 were complexed with negative control siRNA (Thermo Fisher Scientific) or PLK1 siRNA (GE Dharmacon), and incubated with cells in the presence of 5 nM C225.2/PFO^T490A,L491V^. Expression levels of PLK1 mRNA and protein were measured after 24 h by qPCR and western blot respectively, as described below. Cell viability was measured after 48 hours using the WST-1 reagent (Roche) following manufacturer's instructions. All values were normalized to that of control cells treated with 5 nM C225.2/PFO^T490A,L491V^ only.

### qPCR

RNA extraction was performed using the NucleoSpin RNA kit (Clontech) according to manufacturer's instructions. RT-PCR and amplification were performed using the QuantiTect SYBR Green RT-PCR kit (Qiagen) according to manufacturer's instructions on a Roche Lightcycler 480 (Roche). β-actin was used as the housekeeping gene. Primer sequences are listed below. Data were analyzed using the comparative C_T_ method.

PLK1 forward: 5΄-CACCAGCACGTCGTAGGATTC -3΄

PLK1 reverse: 5΄-CCGTAGGTAGTATCGGGCCTC-3΄

β-actin forward: 5΄-GTCTGCCTTGGTAGTGGATAATG-3΄

β-actin reverse: 5΄-TCGAGGACGCCCTATCATGG-3΄

### Western blot

Cells were incubated in lysis buffer (1× LDS sample buffer (Thermo Fisher Scientific) containing 0.1 M DTT (Amresco), Benzonase (1:3000; Sigma) and protease inhibitors (Roche)) for 20 min at 4°C with shaking, followed by manual scraping. Following SDS-PAGE, proteins were transferred to a nitrocellulose membrane using the iBlot Dry Blotting System (Thermo Fisher Scientific) and blocked for 1 h in TBST containing 5% non-fat milk. Incubation was performed with mouse anti-PLK1 (1:100, clone F8, Santa Cruz Biotechnology) at 4°C overnight and HRP-conjugated donkey anti-mouse IgG (1:3000, BioLegend) for one hour at room temperature. Signal was developed using SuperSignal West Pico chemiluminescent substrate (Thermo Fisher Scientific). Antibodies were then stripped using Restore western blot stripping buffer (Thermo Fisher Scientific) and the membrane was re-probed using mouse anti-β-actin (1:15 000, clone AC15, Thermo Fisher Scientific). Band intensities were quantified using ImageJ.

### Uptake assays

A431-d2EGFP cells were seeded at a density of 15 000 cells/well in 96-well plates 16–20 h prior to the experiment. The p19-E18 constructs were incubated with fluorescently labeled siRNA (Seq I) at a 1:1 molar ratio for 30 min at 4°C. The p19-E18/siRNA complexes were then diluted in DMEM containing 10% FBS at varying concentrations and incubated with cells for 0–6 h in a reverse timecourse. For competition experiments, incubation was performed with 20 nM of p19-E18/siRNA complexes and varying concentrations (0–4 μM) of SUMO-E18 in complete media. After 6 h, cells were washed with PBS, trypsinized, neutralized with cold PBSA containing 2% FBS and analyzed on an iQue Screener (IntelliCyt). All liquid handling was performed using an EL406 plate washer (BioTek) and a Freedom EVO 150 liquid handling system (Tecan) to minimize variability. Background from untreated cells were subtracted from all measurements, which were then converted to number of fluorophores using Quantum Alexa Fluor 647 MESF beads following manufacturer's instructions (Bangs Laboratories).

### Model construction

A mathematical model of ordinary differential equations (ODE) (Supplementary Figure S1) was developed describing the trafficking of the siRNA–carrier complex through extracellular, endosomal and cytoplasmic compartments. Multiple species were monitored due to the modular nature of binding (Supplementary Table S2). A net internalization model was employed for receptor-mediated uptake. The siRNA carrier was implemented as a monovalent binder to the receptor for simplification using apparent affinity values. Endosomal release was modeled as a first-order process, the rate of which was fitted from linking the uptake and silencing data of p19-E18. RNA interference was implemented using a simplified model adapted from Bartlett *et al*. (23) (Supplementary Figures S2–S4). Parameters were obtained from the literature or measured experimentally unless stated otherwise (Supplementary Tables S3 and S4). For validation, the model was confirmed to faithfully predict the silencing behavior of the higher affinity p19-E18 clones (Supplementary Figure S5). All simulations were performed using MATLAB (MathWorks). A detailed description and rationale of the mathematical model is provided in Supplementary Data (Supplementary Figures S3–S5).

## RESULTS

### Engineering and characterization of high-affinity p19 clones

**Figure 1.**
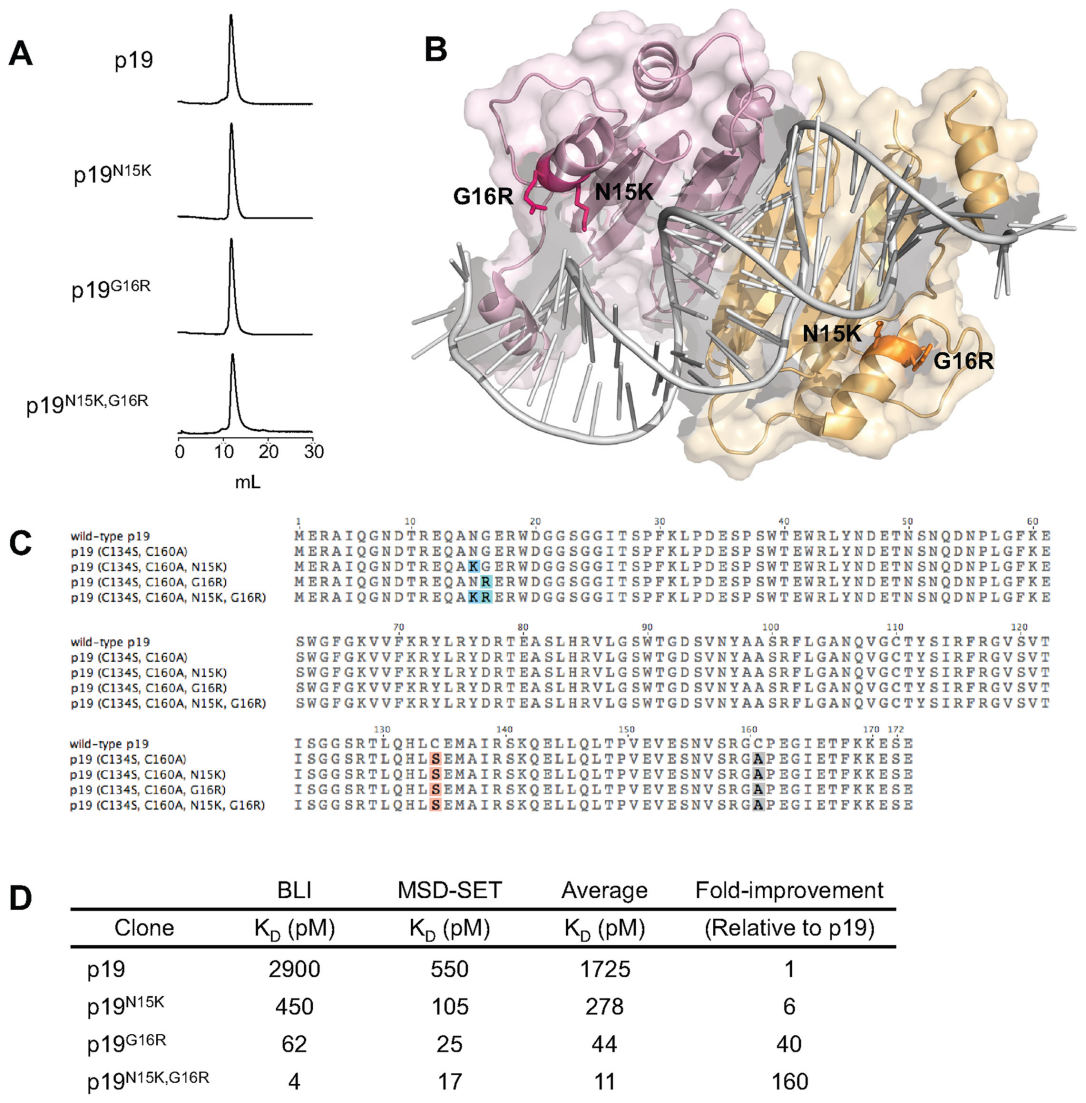
Affinity-matured p19 clones display high affinity and stability. (**A**) SEC analysis of the p19 clones. Thirty μg of each protein was analyzed in PBS at 1 mg/ml. (**B**) Model structure of p19^N15K,G16R^. The highlighted substitutions were introduced into the crystal structure of wild-type p19 (1RPU) using PyMOL. The two monomers forming the homodimer are shown in different shades. (**C**) Sequences of the p19 clones used in this study. Variations from the wild-type sequence are highlighted. (**D**) Affinity measurements using biolayer interferometry (BLI) and soluble equilibrium titrations using MSD technology (MSD-SET). Shown for each method are the averages of two measurements. The results from each method were averaged to calculate the overall fold-improvement in affinity.

To develop p19 into a monodisperse siRNA carrier, we first mutated its solvent-exposed cysteines to non-reactive residues to prevent uncontrolled crosslinking. Wild-type p19 contains three free cysteines, two of which are exposed to solvent (C134, C160) and one embedded within the core (C110). Introducing the C134S and C160A substitutions (24) eliminated multimerization and yielded a monomeric peak when analyzed by size-exclusion chromatography (SEC) (Figure 1A). The clone p19^C134S,C160A^ is herein referred to as ‘p19’.

Next, we increased the affinity of p19 using yeast surface display, a well-established technique for directed evolution. A library of p19 clones was generated by error-prone PCR and displayed on the surface of yeast. The resulting yeast library was subject to multiple rounds of kinetic selections performed at 37°C in 55% mouse serum, to enrich for tighter binders under experimental conditions that mimic an *in vivo* environment. The siRNA antigens used for selections were rotated between three different sequences (Seq F, Seq I and Cy5-labeled AllStars negative control siRNA) to prevent affinity maturation towards a specific sequence. Two out of the three siRNAs contained 2΄OMe modifications at select positions (Seq F and Seq I).

Sequence analysis of the enriched library revealed convergence to a single clone, p19^G16R,D47N^ (19 out of the 20 clones analyzed). The remaining clone outside of this family contained the mutations N15K and I123V. Interestingly, N15K and G16R are in close proximity to the siRNA backbone (Figure 1B), suggesting that the positively charged side chains of Lys and Arg may interact with phosphates on the siRNA backbone. D47N is in an unstructured loop that is not involved in binding, and I123V is at the dimerization interface. To analyze the contribution of each mutation towards binding affinity or protein stability, p19 clones containing different combinations of the aforementioned mutations were expressed solubly. The D47N mutation was found to affect neither soluble expression levels nor binding affinities, and I123V had a destabilizing effect (data not shown). Thus, clones containing either of these mutations were not pursued further.

The p19 clones selected for further analysis contained the substitutions N15K and G16R, individually or in combination (Figure 1C). All three expressed well with comparable yields to p19 (Supplementary Figure S6). The *A*_260_/*A*_280_ ratios of the clones following his-tag purification were between 0.8 and 1.3, indicating that contaminating nucleic acids may be bound to the proteins non-specifically. Anion exchange chromatography (AEX) reduced the *A*_260_/*A*_280_ ratios to between 0.59 and 0.64, effectively stripping away the fugitive nucleic acids. Following purification, all p19 clones eluted as monomeric peaks from SEC without evidence of aggregation, suggesting high stability (Figure 1A).

Finally, the binding affinities of the p19 clones were analyzed using two orthogonal techniques: BioLayer Interferometry (BLI), and a soluble equilibrium titration method utilizing MSD technology (MSD-SET) (Figure 1D). The same siRNA antigen (Seq 7) was used for both analyses to maintain uniformity between assays, which contained 2΄OMe modifications at select positions. The affinity of p19 measured by both methods closely matched values reported in the literature for wild-type p19 (13,25–27), suggesting that binding was not significantly affected by the removal of surface cysteines (as expected) or the modification of select 2΄ hydroxyls. P19 engages the 2΄ hydroxyls at certain positions (12,13), but the remaining network of contacts made with phosphate groups and the end-capping interactions made at the siRNA termini likely maintain binding. Measurements made by both methods showed a consistent trend where p19^N15K,G16R^ had the highest affinity, followed by p19^G16R^, p19^N15K^ and p19. Overall, we successfully created a series of p19 clones with multiple-fold improvements in affinity, up to 160-fold for the tightest binder.

### Development and characterization of targeted, high affinity siRNA carriers

**Figure 2.**
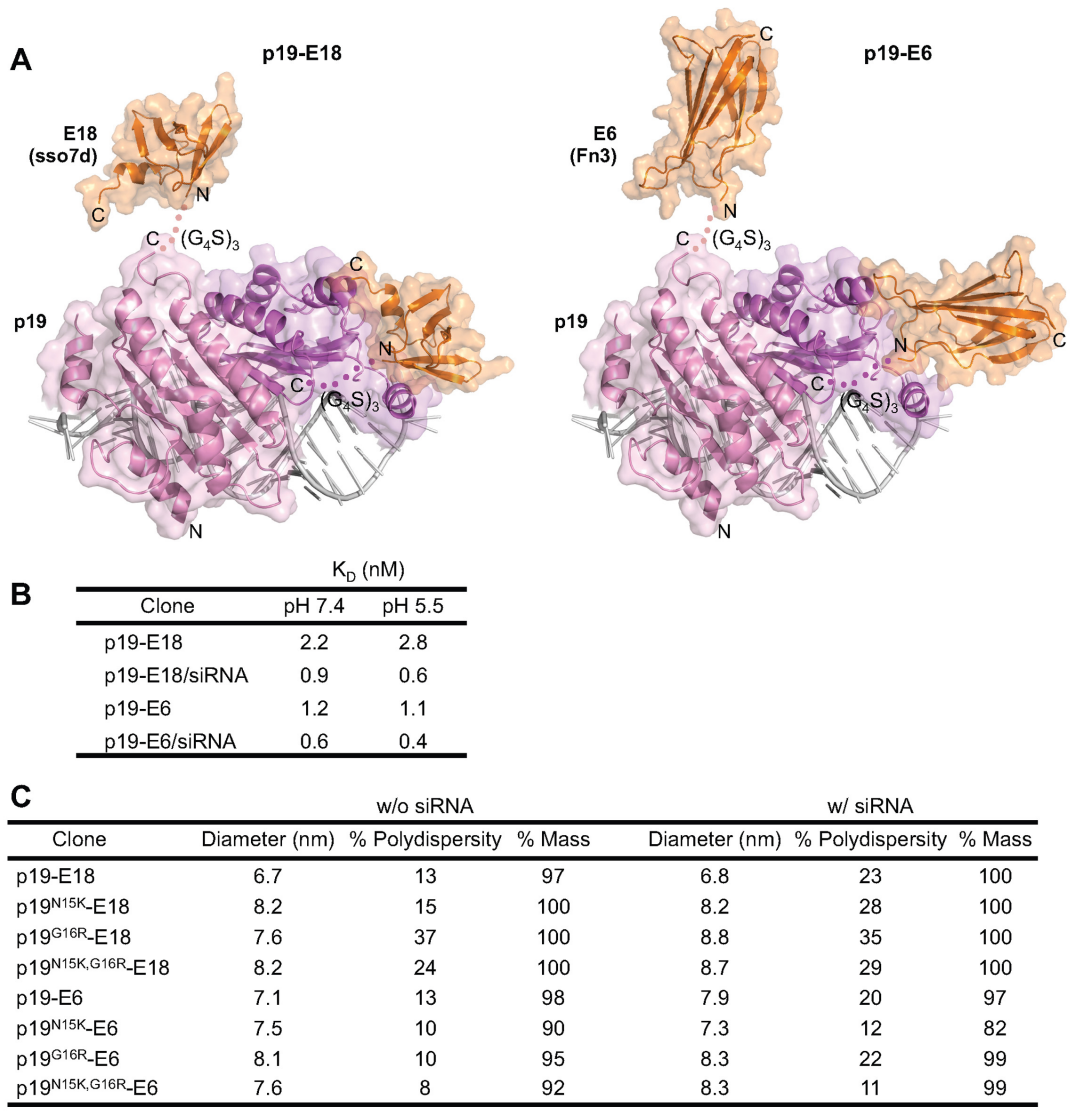
Characterization of EGFR-targeted p19 clones. (**A**) Model structures of the targeted p19 constructs used in this study. Left: E18, an EGFR binder engineered on a modified sso7d scaffold (1SSO) (20), was fused to the C terminus of p19 separated by a (G_4_S)_3_ linker. Right: E6, an EGFR binder engineered on the Fn3 scaffold (1TTG) (28), was fused to the C terminus of p19 separated by a (G_4_S)_3_ linker. (**B**) EGFR affinities of the targeted p19 constructs measured by biolayer interferometry (BLI). Analysis was performed with either empty or siRNA-loaded constructs in citrate-phosphate buffer. Shown are the averages of two independent measurements. (**C**) Size distribution of empty or siRNA-loaded p19 constructs measured by dynamic light scattering (DLS). All samples were analyzed at 5 μM in PBS. Shown are the averages of two independent measurements.

To use the p19-based siRNA carrier in combination with our PFO-based endosome-disrupting agent, which was targeted to EGFR via the antibody Cetuximab (C225), we created two sets of p19 constructs fused to different EGFR binders (Figure 2A). The p19 clones were targeted to the same receptor as the endosome-disrupting agent to maximize the overlap of siRNA and PFO in endosomal compartments. For the same reason, we chose binders that were confirmed not to compete with C225 for receptor binding.

Two separate siRNA carriers were created by genetically linking p19 to two different EGFR binders, which speak to the modularity of the design where p19 can be readily linked with a targeting moiety of choice. The first binder was based on a modified Sso7d scaffold, termed ‘E18’ herein (originally clone E18.6 (20), and the second binder was based on the fibronectin (Fn3) scaffold, termed ‘E6’ herein (originally clone E6.2.6 (28)). Fusing E6 to the C terminus of p19 allowed slightly better silencing compared to its N-terminal counterpart, and thus only C-terminal fusions were considered for further analysis (data not shown). All carrier constructs were pure (Supplementary Figure S7) and eluted as monomeric peaks from SEC (Supplementary Figure S8), which suggested that they were stable and unaggregated.

Next, we characterized the EGFR affinities and size distributions of the carriers following siRNA loading, to confirm that they behave as monodisperse carriers targeting the receptor. Theoretically, the p19 homodimer is capable of binding only one molecule of siRNA at a time, due its caliper-like mode of binding that caps each siRNA termini (13). Thus, we expected that siRNA-induced aggregation was unlikely.

First, the binding affinities of p19-E6 and p19-E18 against EGFR were measured by Biolayer Interferometry (BLI), where a 1:1 binding model was used to obtain apparent affinity values. The analysis was performed at pH 7.4, mimicking extracellular pH, and pH 5.5, mimicking endosomal pH. SiRNA was loaded onto the carriers by mixing at a 1:1 molar ratio (p19-E6/E18 dimer: siRNA) at high concentrations (μM range) to drive loading. Interestingly, complexation with siRNA slightly increased the affinities to EGFR (Figure 2B). This may be due to the siRNA providing further stabilization of the p19-E6/E18 dimer, which is expected to have higher affinities than the monomer due to avidity effects. Binding to EGFR was independent of pH for both carriers, regardless of whether they were loaded with siRNA. P19-E6 and p19-E18 had similar affinities for EGFR, making them functionally equivalent constructs despite being structurally distinct.

Next, we analyzed the size distribution of the targeted carriers by Dynamic Light Scattering (DLS) before and after siRNA loading (Figure 2C). All p19 clones were tested to discern whether the addition of positive charge in the affinity-matured mutants affected potential tendencies for aggregation. The measured diameters of the carrier/siRNA complexes did not significantly differ from those of the unloaded carrier, and were roughly equal to the length of the p19 homodimer (6 nm) or siRNA (7 nm). Polydispersity was constant or slightly higher following siRNA loading, which may be due to the dynamic association and dissociation of siRNA. However, a high polydispersity was expected overall as p19 and the EGFR binder is connected by a flexible peptide linker that can adopt multiple conformations.

We further confirmed that the p19 clones and siRNA form monomeric complexes by SEC (Supplementary Figure S9). Fluorescently labeled siRNA (Seq I) was used for this purpose, to distinguish the siRNA component from the protein component via the dye. The labeled siRNA did not contain any free dye at detectable levels. At both pH 7.4 and pH 5.5, the siRNA eluted together with p19 in a monomeric peak. A small fraction of dissociated siRNA was detected in the starting p19/siRNA complex but gradually disappeared in the affinity-matured p19/siRNA complexes, consistent with the relative binding affinities that were measured. Interestingly, we observed signs of instability with free p19 (without siRNA) at pH 5.5, which were completely absent with the siRNA-bound counterparts at the same pH. This supported the earlier notion that binding to siRNA may further stabilize the p19 dimer.

Overall, our results suggested that the targeted carrier constructs form uniform complexes with siRNA and can subsequently engage EGFR with high affinity.

### Higher carrier affinity against siRNA correlates with more potent *in vitro* silencing

Next, we compared the silencing potency of the different high-affinity carriers in combination with a PFO-based endosome-disrupting agent that was previously reported (C225.2/PFO^T490A,L491V^) (17). Silencing was measured in A431 cells stably transfected with destabilized EGFP (A431-d2EGFP cells) (11). siRNA was loaded onto the carriers as before by mixing, and the endosome-disrupting agent was prepared as described (17).

In contrast to a previously reported system where a p19 construct targeted to the ephrin receptor EphA2 enabled silencing by itself (29), p19-E6/siRNA and p19-E18/siRNA complexes alone did not affect GFP expression in the absence of C225.2/PFO^T490A,L491V^ (Supplementary Figure S10a). This may be due to the targeting of different receptors, whose internalization pathways and intracellular fates may differ. In contrast, the PFO-based endosome-disrupting agent when used alone did cause a dose-dependent reduction in GFP expression. The targeting antibody C225.2 by itself (without PFO^T490A,L491V^) recapitulated this phenomenon, suggesting that C225.2 was responsible for the suppression rather than pore formation by PFO^T490A,L491V^ (Supplementary Figure S10B). We speculate that C225-mediated blockage of EGF growth signaling may affect the expression of GFP from the CMV promoter, which has been reported to be activated by the MEKK1–JNK pathway (30) downstream of EGF (31). To control for this C225.2-mediated effect, GFP expression levels in cells treated with both siRNA and the PFO-based endosome-disrupting agent were normalized to that of cells treated with the latter only.

**Figure 3.**
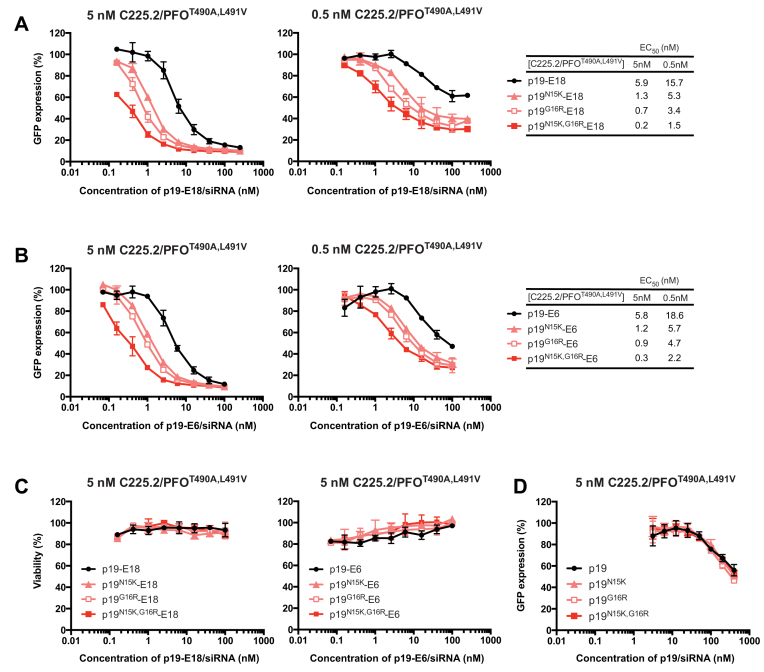
Potent, affinity-dependent silencing mediated by the targeted siRNA carriers. A431-d2EGFP cells were transfected with p19 carriers loaded with GFP siRNA. The concentration of C225.2/PFO^T490A,L491V^ was fixed at either 5 or 0.5 nM. Transfection was performed for 6 h, and GFP expression or cell viability was measured at 24 h. The collected data were normalized to that of control cells treated only with the corresponding concentration of C225.2/PFO^T490A,L491V^. (**A**) Transfection using p19-E18 carriers. Shown is the average of three independent measurements. Normalized data was fitted to a four-parameter logistic curve to obtain the EC_50_ of silencing. (**B**) Identical experiment to (A) but with p19-E6 carriers. (**C**) Cell viability was measured using the WST-1 reagent following the transfection procedures of (A) and (B). (**D**) Identical experiment to (A) but with untargeted p19 carriers. Shown is the average of two independent measurements.

Transfection was performed for 6 h in complete media, after which the media was replaced for an overnight incubation. Potent silencing was observed only when cells were treated with both agents (Figure 3A and B). In addition, silencing was not accompanied with any signs of cytotoxicity (Figure 3C). To strictly compare the effects of siRNA–carrier affinity on silencing potency, the concentration of the endosome-disrupting agent was kept constant to fix the efficiency of endosomal release. The degree of silencing was concentration-dependent on both siRNA and C225.2/PFO^T490A,L491V^, demonstrating that each performed orthogonal roles that were both necessary. Silencing was not observed when negative-control siRNA was used (Supplementary Figure S10C). No significant differences in potency were observed whether E6 or E18 was used as the targeting moiety, consistent with the notion that they are functionally equivalent. Interestingly, we observed that stronger siRNA-carrier affinity consistently led to more potent silencing, with the most potent system achieving an EC_50_ of 230 pM for silencing of GFP. Such a potency *in vitro* is higher than commercial lipofectamine (Supplementary Figure S10d) and among the highest reported for protein-based siRNA delivery methods (32). Furthermore, the complete lack of cytotoxicity created a therapeutic window spanning multiple orders of magnitude.

Removing the targeting moiety from the p19 carriers decreased the silencing efficacy up to 1000-fold (Figure 3D), demonstrating that EGFR-mediated internalization was essential for high potency. This result was expected, as we have previously shown that C225.2/PFO^T490A,L491V^ predominantly permeablizes endosomal membranes following EGFR-mediated internalization (17). Thus, delivery of siRNA is maximal when it can co-localize with PFO^T490A,L491V^ in endosomes most efficiently. The untargeted p19/siRNA complexes were likely taken up by non-specific pinocytosis, an inefficient method for both internalizing into cells and co-localizing into PFO-containing endosomes. At the high concentration ranges where silencing was observed, the potency was independent of affinity. This was likely because all p19 clones were fully associated with siRNA at concentrations significantly higher than their *K*_D_ values.

**Figure 4.**
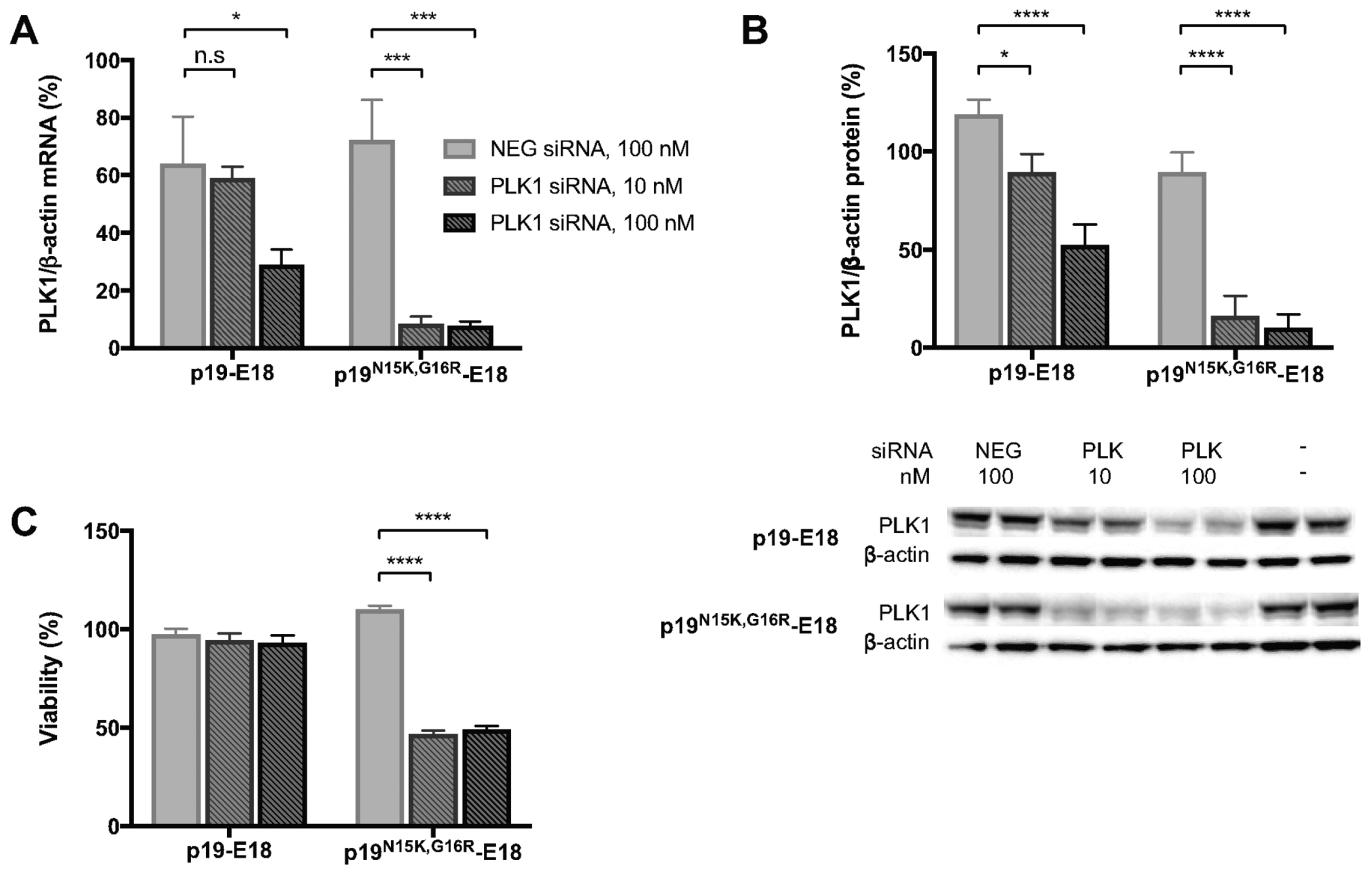
Silencing of endogenous PLK1 is affinity dependent. A431 cells were transfected with p19-E18 or p19-E18^N15K,G16R^ that were loaded with either PLK1 siRNA or negative control (NEG) siRNA. The concentration of C225.2/PFO^T490A,L491V^ was fixed at 5 nM. After 24 h, expression levels of PLK1 (relative to β-actin) were quantified by qPCR for mRNA (**A**) or by western blot for protein (**B**). A representative blot is shown below. (**C**) Cell viability at 48 h after transfection was measured using the WST-1 reagent. All measurements were normalized to that of control cells treated with C225.2/PFO^T490A,L491V^ only. Shown are the averages of three independent experiments ± S.E.M. for each panel. *P < 0.05, ***P < 0.001, ****P < 0.0001 determined by two-way ANOVA with Dunnett's post- test.

Finally, we investigated whether the positive correlation between siRNA-carrier affinity and silencing potency observed with GFP extends also to endogenous genes. To this end, we chose Polo-like kinase 1 (PLK1) as a model target, which is involved in cell division and overexpressed in multiple tumor types (33). As our delivery system targets EGFR, a validated cancer marker (34), silencing of targets that can potentially synergize with EGFR inhibition is a possible application to be explored. As with GFP, p19^N15K,G16R^-E18 produced stronger silencing of PLK1 at both the RNA (Figure 4A) and protein level (Figure 4B) at both concentrations tested, 24 h after transfection. The functional effects of silencing were determined at a gross level by measuring the viability of the culture at 48 h after transfection. Consistent with the relative degrees of silencing, cell viability was significantly lower when p19^N15K,G16R^-E18 was used (Figure 4C). Although p19-E18 did reduce expression of PLK1 it did not affect cell viability, suggesting that either the degree or duration of silencing was insufficient to cause a functional difference.

Overall, the siRNA carriers enabled potent silencing of gene expression in a manner dependent on binding to both siRNA and EGFR.

### siRNA-carrier affinity modulates the intracellular pharmacodynamics of siRNA

**Figure 5.**
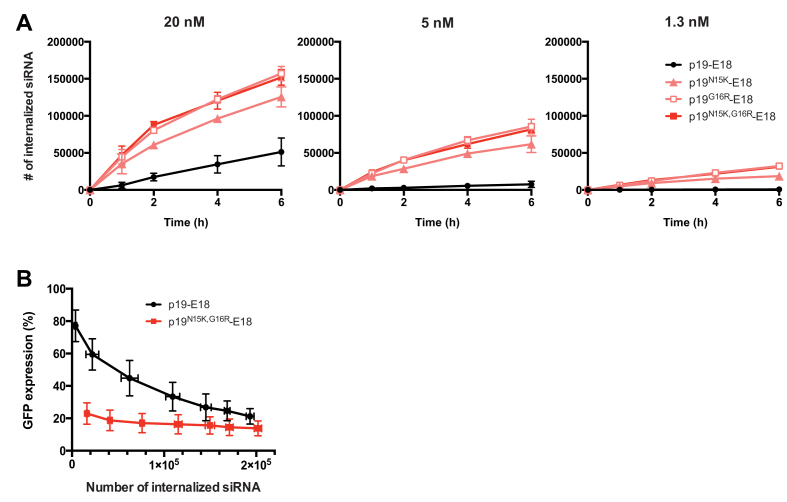
Higher carrier affinity for siRNA improves both uptake into the cell and the downstream efficiency of delivery within the cell. (**A**) The time- and concentration-dependent uptake of siRNA into. A431-d2EGFP cells mediated by each p19-E18 clone. Denoted concentrations are those of the p19-E18/siRNA complex. The p19-E18 clones were loaded with fluorescently labeled siRNA (Seq I), and the background-subtracted fluorescence in cells was converted to number of siRNAs using calibration beads. Shown are the averages of two independent measurements. (**B**) The silencing potencies mediated by p19-E18 and p19^N15K,G16R^-E18 per internalized siRNA. A soluble competitor for EGFR (sumo-E18) was used to titrate the number of siRNA complexes that are internalized. Fluorescently labeled siRNA (Seq I) was used to measure siRNA uptake after 6 h, and GFP siRNA was used to measure GFP knockdown in an analogous setting (Supplementary Figure S12). Shown are the averages of three independent measurements.

We hypothesized that the higher affinity carriers achieved greater silencing because they internalized siRNA more efficiently. Lower affinity carriers have a higher probability of losing their cargo before it can be taken up by receptor-mediated endocytosis. Subsequently, we measured the number of siRNA molecules taken up into A431-d2EGFP cells by each p19-E18 clone using fluorescently-labeled siRNA (Seq I). Our methodology was confirmed to measure internal, but not surface-bound siRNA (Supplementary Figure S11). As expected, higher affinity generally correlated with greater internalization at all concentrations tested (Figure 5A). Uptake eventually saturated with p19^G16R^-E18 and p19^N15K,G16R^-E18, indicating that the internalization limit set by the kinetic properties of the targeting moiety (E18) and receptor (EGFR) were reached. Interestingly, although p19^N15K,G16R^-E18 had consistently produced more potent silencing compared to p19^G16R^-E18 (Figure 3A and B), the number of siRNAs internalized by these carriers were identical over time. This discrepancy suggested that the stronger affinity of p19^N15K,G16R^ may be affecting a step in the delivery process downstream of cellular uptake.

To further probe this observation, we titrated the number of siRNAs being internalized into cells by p19-E18 and p19^N15K,G16R^-E18 and compared the corresponding degrees of silencing. To equalize the number of siRNA and carriers that are taken up, the carrier/siRNA complexes were fixed at a high concentration (20 nM) while a competitor for receptor binding was introduced at increasing concentrations. E18 fused to a SUMO tag (SUMO-E18) was used as the competitor. A large molar excess of SUMO-E18 was required to compete with the p19-E18 carriers, as the binding interaction of the former is monovalent and the latter bivalent. With this setup, both p19-E18 and p19^N15K,G16R^-E18 internalized decreasing numbers of siRNA with increasing concentrations of SUMO-E18 (Supplementary Figure S12A), confirming that the carrier/siRNA complexes were taken up specifically via EGFR. Interestingly, under the same setup but with the addition of a fixed concentration of the PFO-based endosome-disrupting agent, p19-E18/siRNA displayed a gradual decrease in silencing, whereas silencing by p19^N15K,G16R^-E18 was largely unaffected (Supplementary Figure S12B). Plotting the number of internalized siRNA and the corresponding expression levels of GFP highlights that the per-siRNA potency of silencing is higher when siRNA is delivered by the higher affinity carrier (Figure 5B), even when similar numbers of siRNA are taken up into the cell.

**Figure 6.**
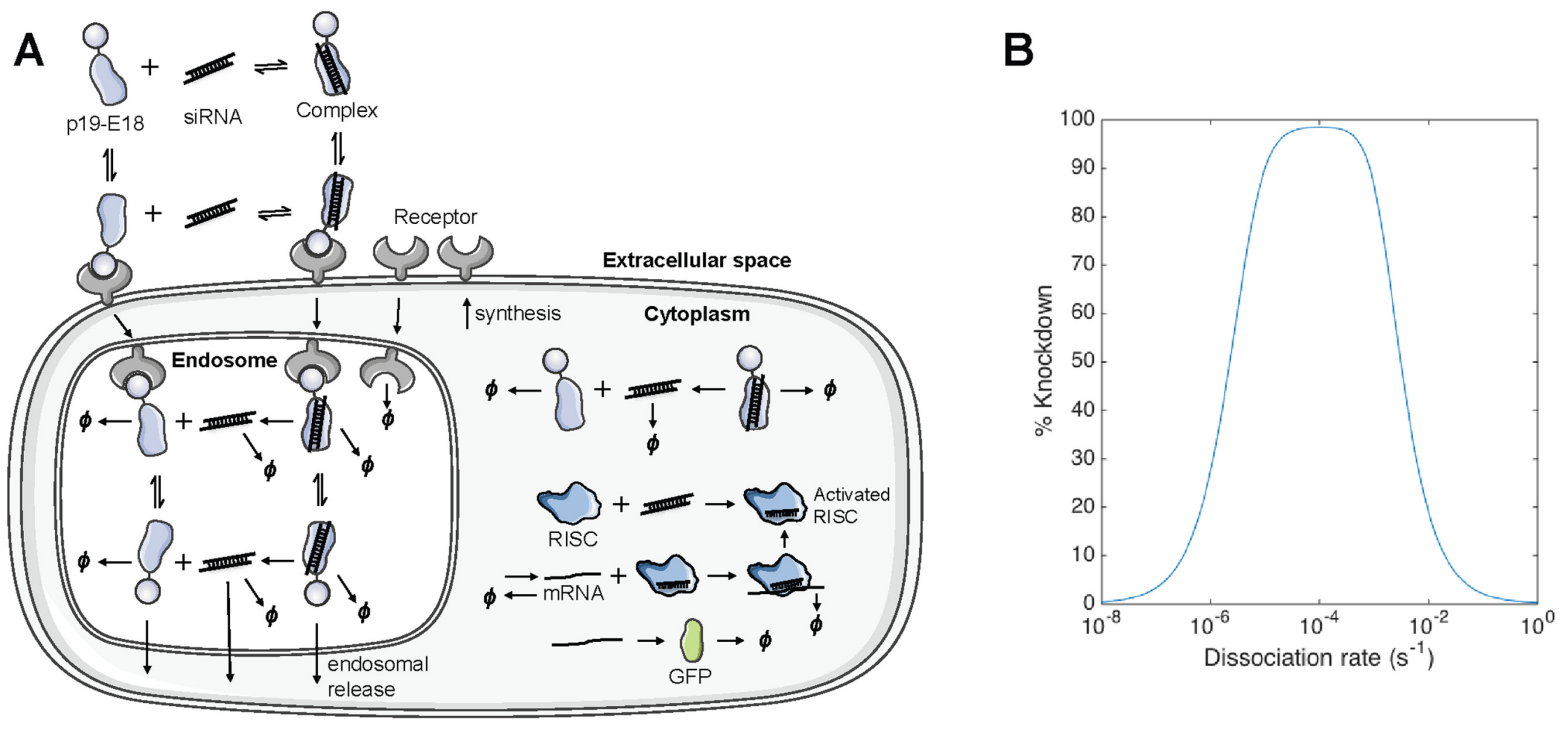
Mathematical modeling predicts the existence of an affinity optimum that maximizes silencing. (**A**) Schematic of mathematical model. Mass action kinetics was used to describe the delivery of siRNA between extracellular, endosomal and cytoplasmic compartments. The concentrations of free siRNA, free carrier and the siRNA-carrier complex were tracked separately in each compartment. Ø indicates degradation. (**B**) The predicted dependence of silencing potency on the dissociation rate between siRNA and its carrier. All other parameters were held constant, including the rate of endosomal release and the initial extracellular concentration of siRNA.

This result initially appeared counterintuitive, as once the siRNA/carrier complex is internalized, stronger binding between the two is expected to hamper siRNA discharge and subsequent loading onto RISC. Furthermore, it raised the question of whether and how far siRNA–carrier affinity could be increased for additional improvements in efficacy. To address these questions, we built a mathematical model of our delivery system and simulated silencing at varying affinities (Figure 6). Briefly, we first created a simplified model for RNA interference using data in the literature correlating the number of cytoplasmic siRNA and silencing (Supplementary Figure S2). Next, the uptake of p19-E18/siRNA complexes was incorporated using a receptor net-internalization model (Supplementary Figure S3). Finally, a first-order rate of endosomal release was determined using the uptake and silencing data of p19-E18 (Supplementary Figure S4). The resulting model was validated by predicting the silencing behavior of the higher affinity carriers (Supplementary Figure S5).

To simplify model simulations, the affinity of the carrier was varied by fixing the on-rate at 1 × 10^5^ M^−1^ s^−1^, a standard estimate for biomolecular interactions, and varying the off-rate only. The modeling results predict that after an initial gain, continually increasing the siRNA-carrier binding strength would indeed eventually decrease silencing (Figure 6b). This simulation suggests the existence of an affinity optimum that maximizes silencing when all other parameters are equal. The eventual decrease in silencing is predicted to stem from siRNA sequestration by p19 (Supplementary Figure S13), mirroring the natural function of p19 as a viral inhibitor of RNA interference in the infected host (35).

Overall, our experimental observations and modeling results suggest that tighter binding between siRNA and its carrier may improve the cellular uptake and intracellular pharmacodynamics of siRNA, but that this trend would not continue indefinitely. As such, we posit that there exists an optimal affinity that maximizes the efficiency of delivery via RNA binding proteins, and propose that this may also hold true for other non-covalent delivery materials.

## DISCUSSION

In this study, we have reported the engineering of ultra-high affinity siRNA binders based on the viral protein p19, which contained the substitutions N15K and G16R that likely engage backbone phosphates. As targeted siRNA carriers, these high affinity p19 clones improved the potency of silencing up to 20-fold, yielding a notably effective system. This improvement was partially due to increased cellular uptake, and also to more efficient delivery to RISC following siRNA internalization. Indeed, higher potencies were achieved when siRNA was loaded onto the higher affinity carrier, even when equal numbers were internalized.

Our experimental findings were unexpected, as high affinity in the intracellular space has generally been deemed undesirable for delivery systems relying on non-covalent packaging of siRNA, in some cases motivating the development of vehicles that decrease affinity in response to environmental cues within the cell (36). In the cytoplasm, inefficient dissociation of the siRNA complex can prevent the release of free siRNA for loading onto RISC. In the endosome, inefficient dissociation may hinder the endosomal escape of siRNA (37). Although the exact mechanisms underlying endosomal escape are not clearly understood for most delivery systems, it is also conceivable that the endosome-disrupting component itself requires liberation, necessitating the delivery particle to dissociate globally. Consequently, with delivery systems that are packaged into a single entity in particular, siRNA–carrier binding strength may be coupled with other parameters in a manner that renders it challenging to investigate independently.

When the affinity-dependence of silencing potency was characterized unambiguously in our delivery system, three separate affinity regimes were identified. In the first regime, low affinity limited the efficient uptake of siRNA, as siRNA and its targeted carrier could not remain stably associated. Thus, increasing the affinity in this regime led to more efficient uptake of siRNA and subsequently more efficient silencing. In the second regime, affinity no longer limited uptake, which was now saturated, but increasing affinity again led to better silencing. In the third regime, siRNA discharge was predicted to become limiting, and increasing affinity correlated with a decreased efficiency of silencing. Our results mirror the experimental observations made with a polyplex delivery system, where increasingly stronger siRNA-polymer binding correlated with an increase in silencing potency followed by an eventual decline *in vitro* and *in vivo* (16). Future studies will be required to validate the experimental and theoretical concepts of affinity-dependence in different models of siRNA delivery.

The exact mechanism of how tighter siRNA-carrier association improves siRNA potency without increasing uptake also requires further investigation. It is unlikely that the efficiency of endosomal escape was altered in our experimental setup, as the endosome-disrupting functionality was provided by a physically separate agent. Also, the pores formed by PFO are 25–30 nm in diameter (38), which are in theory sufficiently large to allow the passage of an entire p19–siRNA complex approximately 8 nm in diameter (Figure 2C). It is possible that siRNA was better protected against intracellular nucleases, either in endolysosomal compartments or the cytoplasm. In particular, while slower unpackaging in the cytoplasm can penalize potency by hindering the release of free siRNA, slower unpackaging in endosomes presents no obvious penalty based on the design of our delivery system. Being able to maintain a higher concentration of intact siRNA for a prolonged period of time in endocytic compartments may conceivably increase the probability of successful escape events.

Multiple improvements reported in this study increase the feasibility of this delivery system functioning *in vivo*. Biophysically, p19s are resistant to siRNA-induced aggregation and capable of high affinity binding to siRNA containing chemically modified 2΄ hydroxyls. Practically, the targeted p19 carriers can be expressed, purified and loaded with siRNA in a straightforward manner that is amenable to large-scale production. Functionally, the ultra-high affinity p19 clones are expected to improve the stability of the siRNA-carrier complex during circulation, providing better protection and targeting capabilities. In addition, the exceptional *in vitro* potency of the delivery system suggests that silencing can be achieved with reasonable doses of siRNA. Finally, the two-component system developed here is simpler than our previously reported system of three components (11), and supported by successful precedents of *in vivo* co-injection strategies. For example, the Dynamic Polyconjugate systems where targeted siRNA (cholesterol-siRNA) was co-administered with co-targeted endosome-disrupting agents (PBAVE-based polymers (39) or melittin-like peptides (40)) achieved efficient silencing in the liver. The non-particulate nature of our delivery system, in combination with its modularity to swap in binders against different targets, can potentially allow efficient targeting of other internal organs or tumors.

Lastly, outside of siRNA delivery, the ultra-high affinity p19 clones reported here may also serve as useful tools for various other biological applications. For example, wild-type p19 has already been used to isolate miRNA or siRNA from biological samples (41–50); inhibit RNAi in plants to enhance yields of recombinant proteins (51–53); or inhibit RNAi in mammalian cells to enhance titers of recombinant adenovirus (54). Rauschhuber *et al*. also demonstrated *in vivo* utility by inhibiting RNAi in a tissue-specific manner (54). Additionally, p19 has been used to stabilize siRNAs in bacteria for recombinant production of siRNA in *E. coli* (55). As these implementations all depend on tight binding to siRNA, the ultra-high affinity p19 clones reported here are expected to further enhance application performance and utility.

## Supplementary Material

Supplementary Data
