# Supplementary material for: Cytosolic delivery of siRNA by ultra-high affinity dsRNA binding proteins
Source: Nucleic Acids Res. 2017 Jun 21;45(13):7602–14. doi: 10.1093/nar/gkx546 (PMC5570165; doi:10.1093/nar/gkx546)
Supplement: Supplementary Data [file gkx546_supp.pdf]

Supplementary Data

Cytosolic delivery of siRNA by ultra-high affinity dsRNA binding proteins

Nicole J. Yang[1,*], Monique J. Kauke[1], Fangdi Sun[2], Lucy F. Yang[2], Katie F. Maass[1], Michael W. Traxlmayr[3], Yao Yu[4], Yingda Xu[4], Robert S. Langer[1,3,5,6], Daniel G. Anderson[1,3,5,6], K. Dane Wittrup[1,2,3]

[1] Department of Chemical Engineering, Massachusetts Institute of Technology, Cambridge, MA 02139, USA
[2] Department of Biological Engineering, Massachusetts Institute of Technology, Cambridge, MA 02139, USA
[3] David H. Koch Institute for Integrative Cancer Research, Massachusetts Institute of Technology, Cambridge, MA 02139, USA
[4] Protein Analytics, Adimab LLC, Lebanon, NH 03766, USA
[5] Harvard-MIT Division of Health Science and Technology, Massachusetts Institute of Technology, Cambridge, MA 02139, USA
[6] Institute for Medical Engineering and Science, Massachusetts Institute of Technology, Cambridge, MA 02139, USA

* To whom correspondence should be addressed. Tel: +1 617 432 6152; Fax: +1 617 432 6153; Email: nicole_yang{at}hms.harvard.edu

Present Address: Nicole J. Yang, Department of Microbiology and Immunobiology, Harvard Medical School, Boston, MA 02115, USA

Table S1. siRNA sequences 2
Table S2. Model variables 3
Table S3. Model parameters (part 1) 4
Table S4. Model parameters (part 2) 6
Table S5. Off-rates for model validation 7

Figure S1. Model equations 8
Figure S2. Model construction: RNA interference 10
Figure S3. Model construction: Receptor-mediated uptake of siRNA 11
Figure S4. Model construction: Endosomal release 11
Figure S5. Model validation 12
Figure S6. Expression yields of affinity-matured p19 clones 13
Figure S7. Non-reduced SDS-PAGE of p19 fusion proteins 13
Figure S8. SEC analysis of p19 fusion proteins 14
Figure S9. SEC analysis of p19/siRNA complexes 15
Figure S10. Silencing controls 16
Figure S11. Trypsinization removes surface-bound siRNA 17
Figure S12. Competitor against receptor binding titrates siRNA uptake 18
Figure S13. Carriers with excessively high affinity sequester siRNA in the cytoplasm 19

Supplementary Methods 20
Supplementary References 21

Table S1. siRNA sequences

siRNA	Source	Target	Sequence*
Seq 7	**	Luc	sense: AAcGcuGGGcGuuAAucAAdTdT—**Biotin**<br>antisense: UUGAUuAACGCCcAGCGUUdTsdT
Seq I	**	Factor VII	sense: **AF647**—GGAucAucucAAGucuuAcdTsdT<br>antisense: GuAAGAcuuGAGAuGAuccdTsdT
Seq F (Cy5.5 labeled)	**	Luc	sense: **Cy5.5**—AAcGcuGGGcGuuAAucAAdTsdT<br>antisense: UUGAUuAACGCCcAGCGUUdTsdT
Seq F (unlabeled)	Integrated DNA Technologies	Luc	sense: AACGCUGGGCGUUAAUCAAdTdT<br>antisense: UUGAUUAACGCCCAGCGUUdTdT
AllStar negative control siRNA (Cy5-labeled)	Qiagen	Negative control	N/A
AllStar negative control siRNA (unlabeled)	Qiagen	Negative control	N/A
Silencer Negative Control #1 siRNA	Thermo Fisher Scientific	Negative control	N/A
siGENOME Human PLK1 siRNA	GE Dharmacon	PLK1 (human)	N/A
GFP Duplex I	GE Dharmacon	GFP	N/A

* Lower case letters indicate bases with 2'OMe modifications.
** Provided by Alnylam Pharmaceutics.

Table S2. Model variables

Compartment	Variable	Description*
Extracellular space (Ex)	S^{Ex}	Free siRNA in the extracellular space
	P^{Ex}	Free carrier in the extracellular space
	SP^{Ex}	siRNA/carrier complex in the extracellular space
	R^{Ex}	Free surface receptor
	PR^{Ex}	Free carrier bound to surface receptor
	SPR^{Ex}	siRNA/carrier complex bound to surface receptor
Endosome (En)	S^{En}	Free siRNA in the endosome
	P^{En}	Free carrier in the endosome
	SP^{En}	siRNA/carrier complex in the endosome
	R^{En}	Free receptor in the endosome
	PR^{En}	Free carrier bound to endosomal receptor
	SPR^{En}	siRNA/carrier complex bound to endosomal receptor
Cytoplasm (Cy)	S^{Cy}	Free siRNA in the cytoplasm
	P^{Cy}	Free carrier in the cytoplasm
	SP^{Cy}	siRNA/carrier complex in the cytoplasm
	$RISC^{Cy}$	Free RISC in the cytoplasm
	$SRISC^{Cy}$	Activated RISC in the cytoplasm
	M^{Cy}	Target mRNA in the cytoplasm
	T^{Cy}	Target protein in the cytoplasm

* All variables are in units of moles.

Table S3. Model parameters (part 1)

Group	Parameter	Description	Value	Source
Receptor (R)	numR	Number of receptors per cell	2.8×10^{6}	(1)
	R_0^{Ex}	Initial concentration of surface receptors	7.0×10^{-10} M	Calculated[1]
	R_0^{En}	Initial concentration of endosomal receptors	6.1×10^{-4} M	Calculated from mass balance[2]
	k_{int}^{R}	Internalization rate	4.8×10^{-5} s^{-1}	Measured experimentally
	k_{syn}^{R}	Receptor synthesis rate	3.3×10^{-14} s^{-1}	Calculated from mass balance[3]
	k_{deg}^{R}	Degradation rate of receptors in the endosome	3.7×10^{-5} s^{-1}	(2)
Degradation	$k_{\text{deg}}^{S,En}$	Degradation rate of free siRNA (S) in the endosome	1.4×10^{-4} s^{-1}	(3)
	$k_{\text{deg}}^{P,En}$	Degradation rate of free carrier (P) in the endosome	1.0×10^{-4} s^{-1}	(4)
	$k_{\text{deg}}^{SP,En}$	Degradation rate of the siRNA/carrier complex (SP) in the endosome	1.0×10^{-4} s^{-1}	Set to equal $k_{\text{deg}}^{P,En}$ [4]
	$k_{\text{deg}}^{S,Cy}$	Degradation rate of free siRNA in the cytoplasm	5.1×10^{-4} s^{-1}	(5, 6)
	$k_{\text{deg}}^{P,Cy}$	Degradation rate of free carrier in the cytoplasm	4.2×10^{-6} s^{-1}	(7)
	$k_{\text{deg}}^{SP,Cy}$	Degradation rate of the siRNA/carrier complex in the cytoplasm	4.2×10^{-6} s^{-1}	Set to equal $k_{\text{deg}}^{P,Cy}$ [4]
Endosomal release	$k_{release}$	Rate of endosomal release	3.0×10^{-5} s^{-1}	Locally fitted
Expression of target protein (T)	M_0^{Cy}	Initial concentration of target mRNA (M) in the cytoplasm	7.3×10^{-10} M	(8)
	T_0^{Cy}	Initial concentration of target protein (T) in the cytoplasm	2.1×10^{-7} M	Measured experimentally
	k_{syn}^{M}	Synthesis rate of target mRNA in the cytoplasm	1.1×10^{-15} M^{1}s^{-1}	(7, 9)
	k_{deg}^{M}	Degradation rate of target mRNA in the cytoplasm	1.5×10^{-6} s^{-1}	Calculated from mass balance[5]
	k_{syn}^{T}	Synthesis rate of target protein in the cytoplasm	2.7×10^{-2} s^{-1}	Calculated from mass balance[6]
	k_{deg}^{T}	Degradation rate of target protein in the cytoplasm	9.7×10^{-5} s^{-1}	(10)
RNAi	$RISC_0^{Cy}$	Initial concentration of free RISC in the cytoplasm	3.2×10^{-9} M	(3)
	k_{on}^{S-RISC}	Association rate of free siRNA and RISC	3.3×10^{1} M^{-1}s^{-1}	(3)
	$k_{on}^{S-RISC-M}$	Association rate of activated RISC and mRNA	2.9×10^{7} M^{-1}s^{-1}	Locally fitted
Compartment volumes	V_{Ex}	Extracellular volume	1×10^{-4} L	Specified experimentally
	V_{En}	Endosomal volume	1×10^{-14} L	(11)
	V_{Cy}	Cytosolic volume	3×10^{-12} L	(12)
Cell number	Z	Total number of cells	1.5×10^{4}	Specified experimentally

1. $R_0^{Ex} = \frac{numR \cdot Z}{N_A \cdot V_{Ex}}$ where N_A is Avogadro's constant

2. $R_0^{En} = \frac{k_{int}^{R} \cdot R_0^{Ex} \cdot V_{Ex}}{k_{deg}^{R} \cdot Z \cdot V_{En}}$

3. $k_{syn}^{R} = k_{int}^{R} \cdot R_0^{Ex}$

4. The degradation rate of the siRNA-carrier complex was set to equal that of the more stable component (i.e., the more stable species protects the lesser stable species from degradation, as observed with p19 and siRNA (13)).

5. $k_{deg}^{M} = \frac{k_{syn}^{M}}{M_{0}^{Cy}}$

6. $k_{syn}^{T} = \frac{k_{deg}^{T} \cdot T_{0}^{Cy}}{M_{0}^{Cy}}$

Table S4. Model parameters (part 2)

Parameter	Description	Value[1]
$k_{on}^{SP,Ex}$	Association rate of siRNA and carrier in the extracellular space	1.0×10^{5} $M^{-1}s^{-1}$ (14)
$k_{off}^{SP,Ex}$	Dissociation rate of siRNA and carrier in the extracellular space	Varied (part of simulation)
$k_{off}^{SP,En}$	Dissociation rate of siRNA and carrier in the endosome	Set to equal $k_{off}^{SP,Ex}$ [2]
$k_{off}^{SP,Cy}$	Dissociation rate of siRNA and carrier in the cytoplasm	Set to equal $k_{off}^{SP,Ex}$ [2]
$k_{on}^{S-PR,Ex}$	Association rate of siRNA and receptor-bound carrier in the extracellular space	Set to equal $k_{on}^{SP,Ex}$ [3]
$k_{off}^{S-PR,Ex}$	Dissociation rate of siRNA and receptor-bound carrier in the extracellular space	Set to equal $k_{off}^{SP,Ex}$ [3]
$k_{off}^{S-PR,En}$	Dissociation rate of siRNA from the receptor-bound complex in the endosome	Set to equal $k_{off}^{SP,En}$ [3]
$k_{on}^{PR,Ex}$	Association rate of the carrier and receptor in the extracellular space	1.8×10^{5} $M^{-1}s^{-1}$
$k_{off}^{PR,Ex}$	Dissociation rate of carrier and receptor in the extracellular space	4.0×10^{-4} s^{-1}
$k_{on}^{PR,En}$	Association rate of the carrier and receptor in the endosome	2.0×10^{5} $M^{-1}s^{-1}$
$k_{off}^{PR,En}$	Dissociation rate of the carrier and receptor in the endosome	4.0×10^{-4} s^{-1}
$k_{on}^{SP-R,Ex}$	Association rate of the complex and receptor in the extracellular space	1.6×10^{5} $M^{-1}s^{-1}$
$k_{off}^{SP-R,Ex}$	Dissociation rate of the complex and receptor in the extracellular space	1.5×10^{-4} s^{-1}
$k_{on}^{SP-R,En}$	Association rate of the complex and receptor in the endosome	4.1×10^{5} $M^{-1}s^{-1}$
$k_{off}^{SP-R,En}$	Dissociation rate of the complex and receptor in the endosome	2.4×10^{-4} s^{-1}

1. Measured experimentally unless otherwise noted. The extracellular space was modeled as a pH 7.4 environment, and the endosome pH 5.5.
2. Based on compartment pH. The affinity of p19 does not significantly differ between the two pHs (15).
3. As siRNA loading did not affect receptor binding by the targeted carrier (**Figure 2b**), we assumed that p19 and the EGFR binder function independently within the targeted carrier and that siRNA binding kinetics would not be affected by the carrier's association state to EGFR.

Table S5. Off-rates for model validation

Clone	Off-rate (s^{-1})	Fold difference (Relative to p19)
p19	7.2×10^{-2}	1
$p19^{N15K}$	1.2×10^{-2}	6
$p19^{G16R}$	1.8×10^{-3}	40
$p19^{N15K,G16R}$	4.4×10^{-4}	160

The off-rates were estimated as described in Model Construction: Receptor-mediated uptake of siRNA.

Figure S1. Model equations

$$\frac{dS^{Ex}}{dt} = -k_{on}^{SP,Ex} \cdot S^{Ex} \cdot P^{Ex} + k_{off}^{SP,Ex} \cdot SP^{Ex} - k_{on}^{SPR,Ex} \cdot S^{Ex} \cdot PR^{Ex} + k_{off}^{SPR,Ex} \cdot SPR^{Ex}$$

$$\frac{dP^{Ex}}{dt} = -k_{on}^{SP,Ex} \cdot S^{Ex} \cdot P^{Ex} + k_{off}^{SP,Ex} \cdot SP^{Ex} - k_{on}^{PR,Ex} \cdot P^{Ex} \cdot R^{Ex} + k_{off}^{PR,Ex} \cdot PR^{Ex}$$

$$\frac{dSP^{Ex}}{dt} = k_{on}^{SP,Ex} \cdot S^{Ex} \cdot P^{Ex} - k_{off}^{SP,Ex} \cdot SP^{Ex} - k_{on}^{SPR,Ex} \cdot SP^{Ex} + k_{off}^{SPR,Ex} \cdot SPR^{Ex}$$

$$\frac{dR^{Ex}}{dt} = k_{syn}^{R} - k_{int}^{R} \cdot R^{Ex} - k_{on}^{PR,Ex} \cdot P^{Ex} \cdot R^{Ex} + k_{off}^{PR,Ex} \cdot PR^{Ex} - k_{on}^{SPR,Ex} \cdot SP^{Ex} \cdot R^{Ex} + k_{off}^{SPR,Ex} \cdot SPR^{Ex}$$

$$\frac{dPR^{Ex}}{dt} = -k_{int}^{R} \cdot PR^{Ex} + k_{on}^{PR,Ex} \cdot P^{Ex} \cdot R^{Ex} - k_{off}^{PR,Ex} \cdot PR^{Ex} - k_{on}^{S-PR,Ex} \cdot S^{Ex} \cdot PR^{Ex} + k_{off}^{S-PR,Ex} \cdot SPR^{Ex}$$

$$\frac{dSPR^{Ex}}{dt} = -k_{R}^{int} \cdot SPR^{Ex} + k_{on}^{SP-R,Ex} \cdot SP^{Ex} \cdot R^{Ex} - k_{off}^{SP-R,Ex} \cdot SPR^{Ex} + k_{on}^{S-PR,Ex} \cdot S^{Ex} \cdot PR^{Ex} - k_{off}^{S-PR,Ex} \cdot SPR^{Ex}$$

$$\frac{dS^{En}}{dt} = k_{off}^{SP,En} \cdot SP^{En} + k_{off}^{SP-R,En} \cdot SPR^{En} - k_{deg}^{S,En} \cdot S^{En} - k_{release} \cdot S^{En}$$

$$\frac{dP^{En}}{dt} = k_{off}^{SP,En} \cdot SP^{En} - k_{on}^{PR,En} \cdot P^{En} \cdot R^{En} + k_{off}^{PR,En} \cdot PR^{En} - k_{deg}^{P,En} \cdot P^{En} - k_{release} \cdot P^{En}$$

$$\frac{dSP^{En}}{dt} = -k_{off}^{SP,En} \cdot SP^{En} - k_{on}^{SP-R,En} \cdot SP^{En} \cdot R^{En} + k_{off}^{SP-R,En} \cdot SPR^{En} - k_{deg}^{SPR,En} \cdot SPR^{En} - k_{release} \cdot SPR^{En}$$

$$\frac{dR^{En}}{dt} = k_{int}^{R} \cdot R^{Ex} \frac{V_{Ex}}{Z \cdot V_{En}} - k_{on}^{PR,En} \cdot P^{En} \cdot R^{En} + k_{off}^{PR,En} \cdot PR^{En} - k_{on}^{SP-R,En} \cdot SP^{En} \cdot R^{En} + k_{off}^{SP-R,En} \cdot SPR^{En} - k_{deg}^{R} \cdot R^{En} + k_{deg}^{P,En} \cdot PR^{En} + k_{deg}^{SP,En} \cdot SPR^{En}$$

$$\frac{dPR^{En}}{dt} = k_{int}^{R} \cdot PR^{Ex} \frac{V_{Ex}}{Z \cdot V_{En}} + k_{on}^{PR,En} \cdot P^{En} \cdot R^{En} - k_{off}^{PR,En} \cdot PR^{En} + k_{off}^{S-PR,En} \cdot SPR^{En} - k_{deg}^{PR,En} \cdot PR^{En}$$

$$\frac{dSPR^{En}}{dt} = k_{int}^{R} \cdot SPR^{Ex} \frac{V_{Ex}}{Z \cdot V_{En}} + k_{on}^{SP-R,En} \cdot SP^{En} \cdot R^{En} - k_{off}^{SP-R,En} \cdot SPR^{En} - k_{off}^{S-PR,En} \cdot SPR^{En} - k_{deg}^{SPR,En} \cdot SPR^{En}$$

$$\frac{dS^{Cy}}{dt} = k_{release} \cdot S^{En} \cdot \frac{V_{En}}{V_{Cy}} + k_{off}^{SP,Cy} \cdot SP^{Cy} - k_{on}^{S-RISC} \cdot S^{Cy} \cdot RISC^{Cy} - k_{deg}^{S,Cy} \cdot S^{Cy}$$

$$\frac{dP^{Cy}}{dt} = k_{release} \cdot P^{En} \cdot \frac{V_{En}}{V_{Cy}} + k_{off}^{SP,Cy} \cdot SP^{Cy} - k_{deg}^{P,Cy} \cdot P^{Cy}$$

$$\frac{dSP^{Cy}}{dt} = k_{release} \cdot SP^{En} \cdot \frac{V_{En}}{V_{Cy}} - k_{off}^{SP,Cy} \cdot SP^{Cy} - k_{deg}^{SP,Cy} \cdot SP^{Cy}$$

Figure S1. Model equations (continued)

$$\frac{dRISC^{Cy}}{dt} = -k_{on}^{S-RISC} \cdot S^{Cy} \cdot RISC^{Cy}$$

$$\frac{dSRISC^{Cy}}{dt} = k_{on}^{S-RISC} \cdot S^{Cy} \cdot RISC^{Cy}$$

$$\frac{dM^{Cy}}{dt} = k_{syn}^{M} - k_{deg}^{M} \cdot M^{Cy} - k_{on}^{S-RISC-M} \cdot SRISC^{Cy} \cdot M^{Cy}$$

$$\frac{dT^{Cy}}{dt} = k_{syn}^{T} \cdot M^{Cy} - k_{deg}^{T} \cdot T^{Cy}$$

Model construction

RNA interference. The model for RNA interference developed here was adapted from Bartlett et al. (3) to create a simplified representation. As with the former model, the total number of RISCs available to form activated RISCs was assumed to remain constant, and dissociation of activated RISC was chosen to be negligible (3). We additionally assumed that dissociation of the activated RISC/mRNA complex is negligible and that the target mRNA is immediately cleaved to liberate RISC. The association rate between activated RISC and mRNA was fitted using experimental data from the literature correlating the number of siRNAs in the cytoplasm and the resulting degree of knockdown (**Figure S2**).

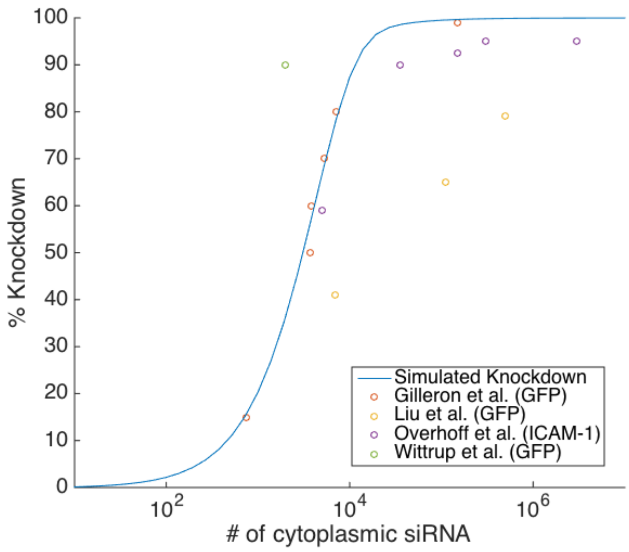

Figure S2. Simulation result for RNA interference. Open circles indicate experimental data, and the solid line represents simulation results.

Receptor-mediated uptake of siRNA. Receptor-mediated internalization implemented by Hackel et al. (16) was simplified to create a net-internalization model capturing the essence of receptor-mediated uptake. As the targeting Fn3 used in this study (clone E6) was demonstrated not to affect surface EGFR levels (16), we assumed that surface EGFR levels are maintained at steady-state throughout the simulation and binding of the targeted carrier does not affect the rate of internalization. Non-specific uptake of siRNA was negligible in the dynamic concentration range (**Figure 3d**), and receptor-bound carriers were allowed to capture free siRNA as observed on the yeast surface. To represent the media change that was performed after 6 hours during the transfection experiments, the concentrations of free species in the extracellular space were set to zero at 6 hours. This effectively removed the siRNA source in the system.

To simplify model simulations, the association rate of the carriers were fixed at 1×10^5 $M^{-1}s^{-1}$ (14), such that their affinity was varied by changing the dissociation rate only. With this on-rate, an effective off-rate was fitted for p19-E18 using experimentally measured uptake kinetics in Figure 4a (**Figure S3**). The fitted off-rate closely matched experimentally reported values (17), although the effective affinity of p19 calculated in this manner was lower than measured values. We speculate that the strong temperature-dependence of p19's affinity or the presence of a nucleic acid competitor in the culture medium may have caused this discrepancy.

Model construction (continued)

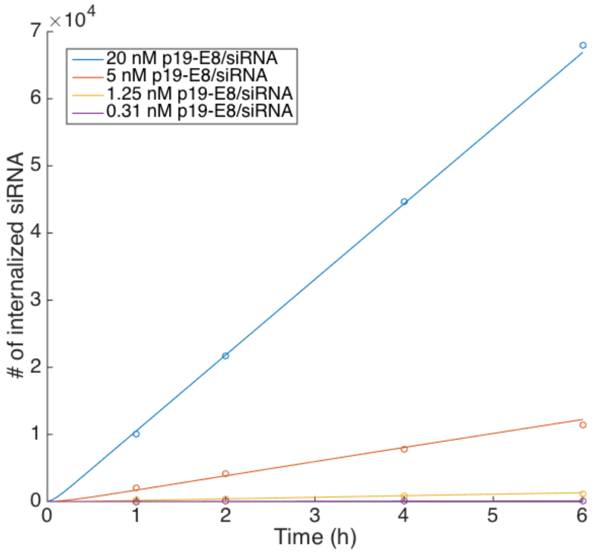

Figure S3. Receptor-mediated uptake of the p19-E18/siRNA complex. Open circles represent experimental data, and solid lines represent simulation results.

Endosomal release. A first-order rate of endosomal release was fitted using the uptake and silencing data of p19-E18. In the absence of experimental evidence suggesting otherwise, we assumed that the rate of escape is equal for all species. This assumption is consistent with the PFO pore (25-30 nm (18)) being larger than all three free species (6-8 nm; **Figure 2c**). Considering competition from multiple intracellular proteins, including nucleases, which contain binding domains for dsRNA (19), we assumed that siRNA rebinding to its carrier is unlikely once dissociated inside the cell. The resulting simulation dependably recapitulated the qualitative behavior of silencing (**Figure S4**).

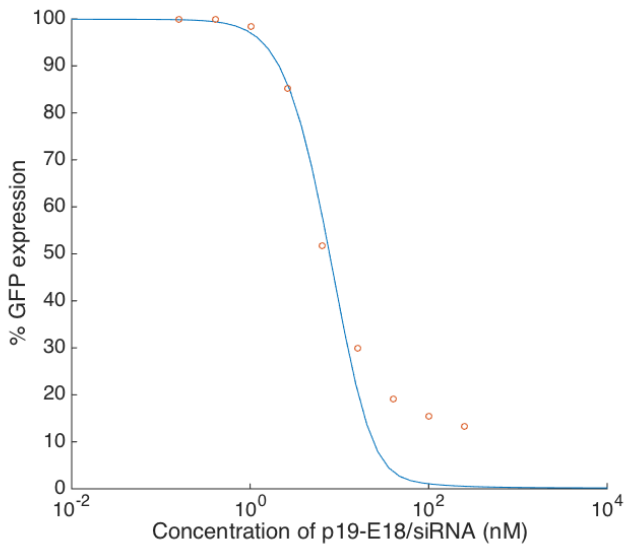

Figure S4. Simulation result for RNA silencing by p19-E18. Open circles represent experimental data, and solid lines represent simulation results.

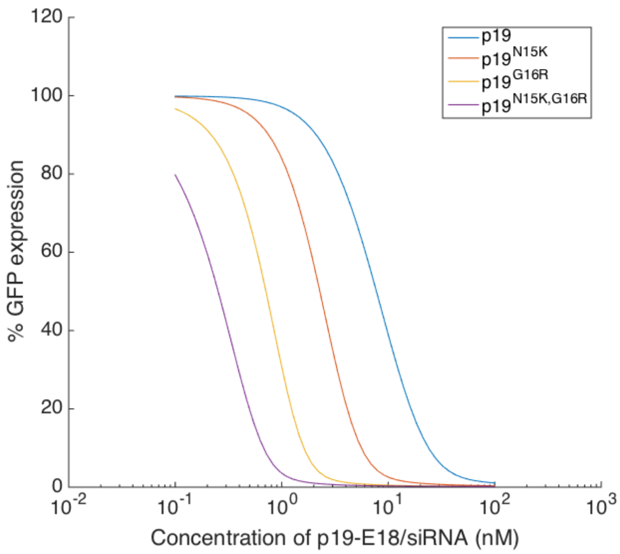

Figure S5. Model predictions of silencing by the higher affinity carriers. Model validation performed by predicting the silencing behavior of the higher affinity carriers. The off-rates of the higher affinity p19 clones were extrapolated based on experimentally measured fold-differences in affinity (**Supplementary Table S5**). Silencing simulated using these off-rates and experimental data (**Figure 3a**) were in good agreement, with the silencing potencies continually improving with higher affinity.

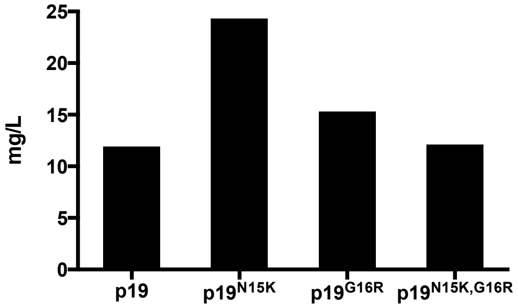

Figure S6. Expression yields of affinity-matured p19 clones. The p19 clones were expressed in *E. coli* as sumo-fusions and purified as described in Materials and Methods. The yields reported here are from a single batch.

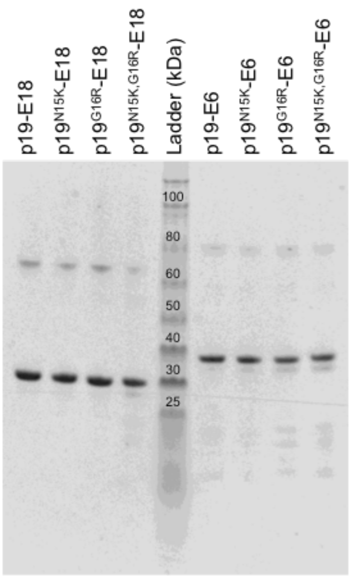

Figure S7. Non-reduced SDS-PAGE of p19 fusion proteins. The expected molecular weight of the p19-E18 and p19-E6 monomers are 27 kDa and 31 kDa, respectively. Dimerization may occur when the embedded cysteine in p19 is exposed following denaturation.

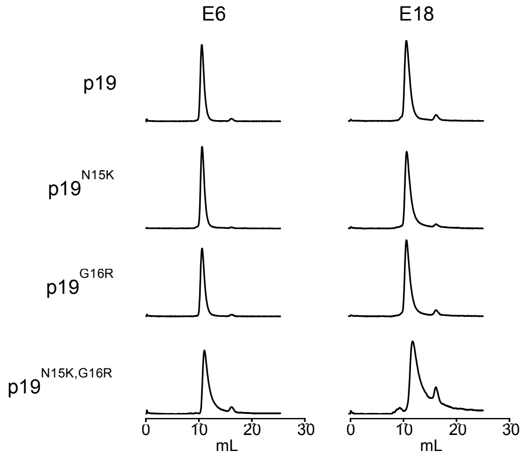

Figure S8. SEC analysis of p19 fusion proteins. For each construct, the targeting moiety is indicated at the top and the p19 moiety on the left. 30 μg of each construct was analyzed as described in Materials and Methods.

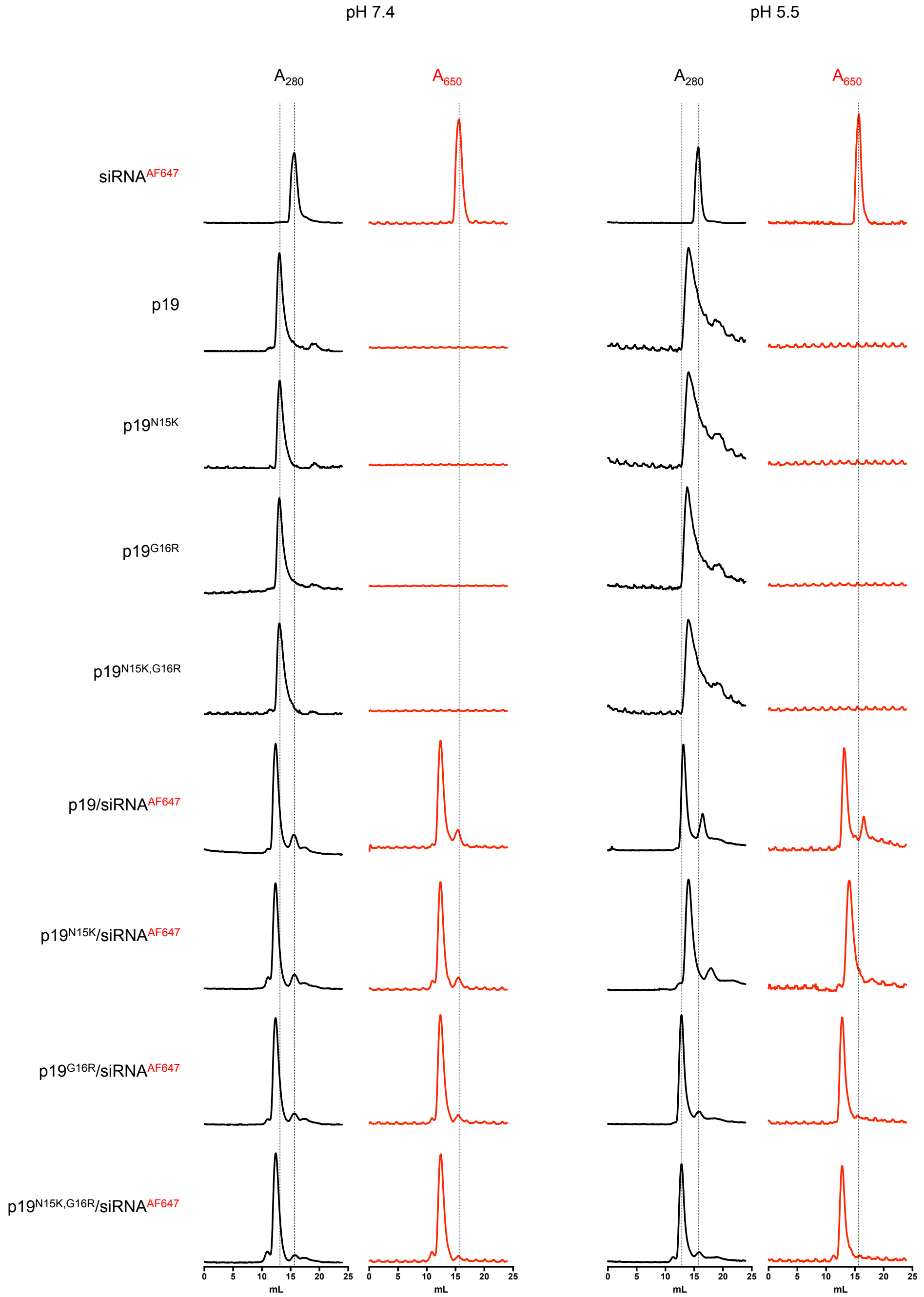

Figure S9. SEC analysis of p19/siRNA complexes. SUMO-tagged p19 clones were complexed with siRNA labeled with Alexa Fluor 647 (Seq I) for 30 min on ice, then analyzed on a Superdex 200 Increase 10/300 GL column in PBS at the indicated pH. The protein and siRNA components were tracked by absorbance at 280 nm and 650 nm, respectively.

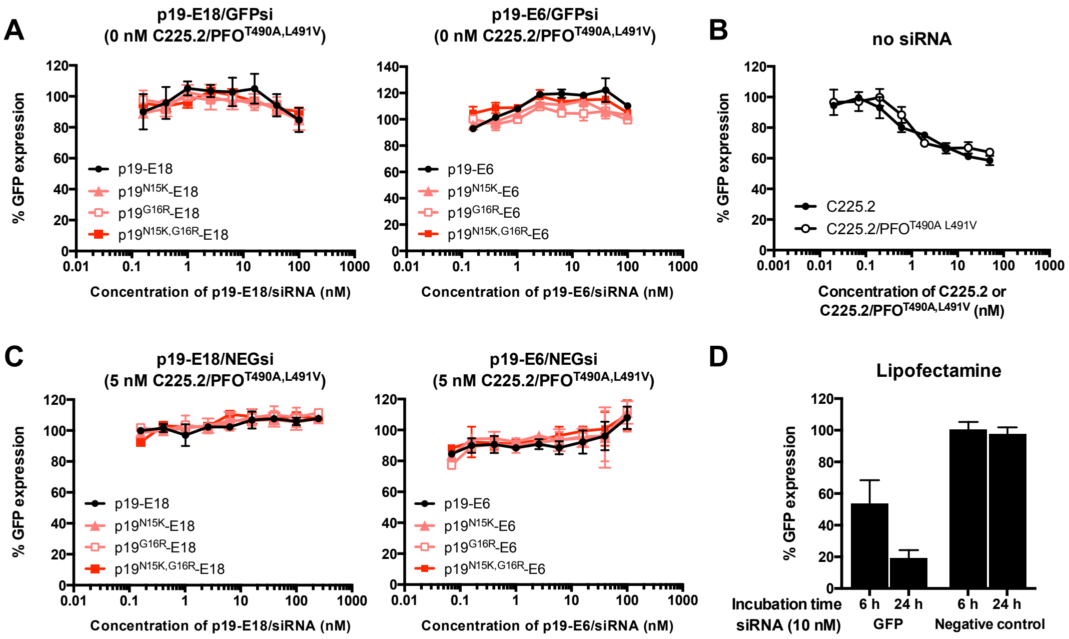

Figure S10. Silencing controls. All experiments were performed with A431-d2EGFP cells. Unless otherwise noted, cells were treated with the respective constructs for 6 hours, then with fresh media for 18 hours. **(a)** Cells were treated with p19-E18 or p19-E6 carriers loaded with GFP siRNA. **(b)** Cells were treated with the targeted, PFO-based endosome-disrupting agent C225.2/PFO^{T490A,L491V} (20) or the targeting antibody C225.2 alone, without PFO^{T490A,L491V}. **(c)** Cells were treated with p19-E18 or p19-E6 carriers loaded with negative-control siRNA. The concentration of C225.2/PFO^{T490A,L491V} was fixed at 5 nM. **(d)** Cells were transfected using the Lipofectamine RNAiMAX reagent following manufacturer's instructions. Cells were either incubated with the lipid-siRNA complexes for 6 hours then with fresh media for 18 hours, or with the lipid-siRNA complexes for 24 hours without changing the media.

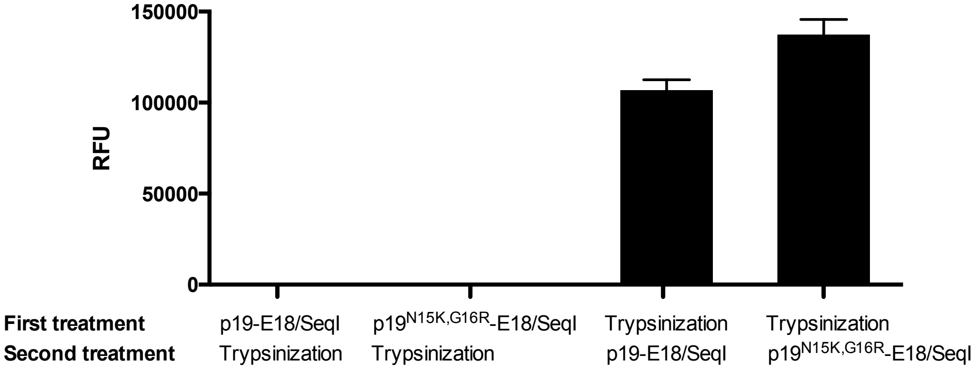

Figure S11. Trypsinization removes surface-bound siRNA. Fluorescently labeled siRNA (Seq I) was loaded onto the carriers p19-E18 or p19^{N15K,G16R}-E18, and incubated with the adherent A431-d2EGFP cell line on ice to prevent endocytosis (1 hour at 20 nM). The incubation was performed either before or after trypsinizing the cells, followed by flow cytometry. Washes were performed between steps with cold PBSA. The trypsinization step completely removed surface-bound siRNA from both carriers (**columns 1-2**), which may be due to potential cleavage of p19. It is unknown whether trypsin stripped the surface-bound carriers as well. The carriers/siRNA complex bound to cells as expected if not exposed to trypsin (**columns 3-4**).

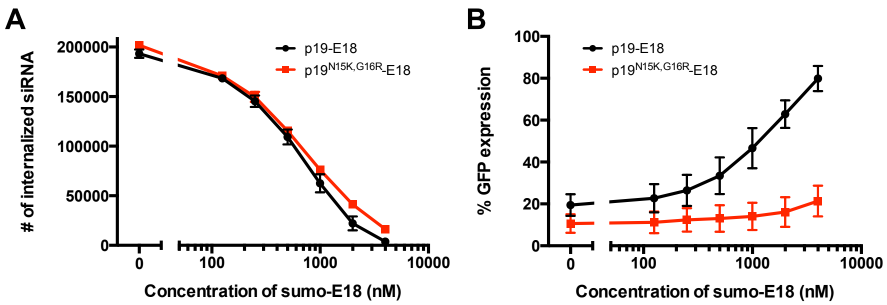

Figure S12. Competitor against receptor binding titrates siRNA uptake. Both experiments were performed in A431-d2EGFP cells. **(a)** Fluorescently labeled siRNA (Seq I) was loaded onto the carriers p19-E18 or $p19^{N15K,G16R}$-E18 by mixing. 20 nM of the respective carrier/siRNA complexes were incubated with cells for 6 hours at 37°C, in the presence of varying concentrations of sumo-E18. The number of internalized siRNA was then quantified as described in Materials and Methods. **(b)** GFP siRNA was loaded onto the carriers p19-E18 or $p19^{N15K,G16R}$-E18 by mixing. 20 nM of the respective carrier/siRNA complexes were incubated with cells for 6 hours at 37°C, in the presence of varying concentrations of sumo-E18 and a fixed concentration of the PFO-based endosome-disrupting agent (C225.2/$PFO^{T490A,L491V}$, 5 nM). The constructs were then replaced with fresh media, and GFP expression was measured at 24 hours.

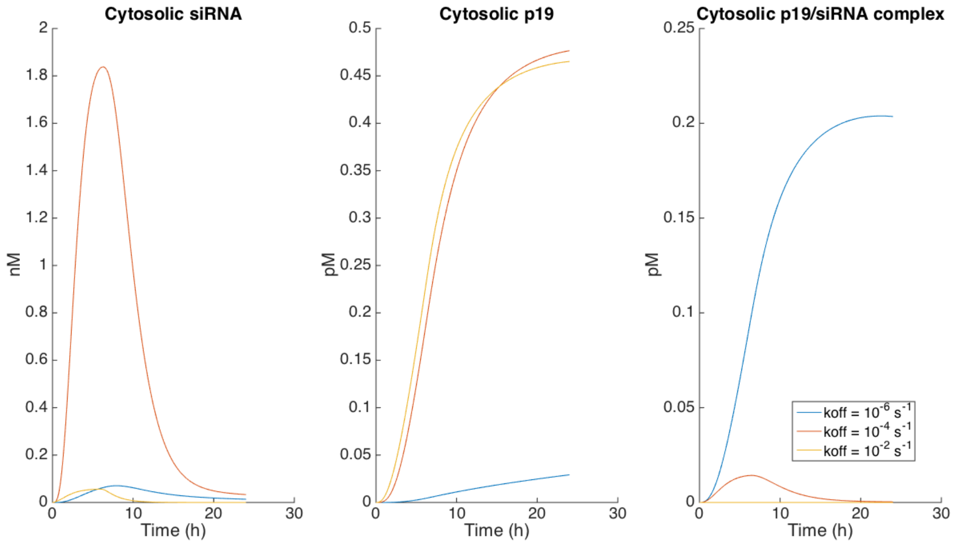

Figure S13. Simulated changes in cytosolic concentrations from three representative off-rates. Carriers with very high affinity ($k_{off}^{SP} = 10^{-6} s^{-1}$) sequester siRNA in the cytoplasm, preventing the release of free siRNA and its subsequent incorporation into RISC. In contrast, carriers with very low affinity ($k_{off}^{SP} = 10^{-2} s^{-1}$) are unable to internalize siRNA.

Supplementary Methods

1. Expression and purification of p19

P19 expression. The p19, p19-E6 and p19-E18 constructs were cloned into the vector pE-SUMO (LifeSensors) and transformed into chemically competent Rosetta 2 (DE3) *E. coli* (Novagen). An overnight starter culture was diluted 1:100 into Terrific Broth (TB) containing antibiotics (50 mg/L kanamycin and 34 mg/L chloramphenicol) and incubated at 37°C for several hours. When the OD_{600} reached 1.9 – 2.1, the culture was induced with 1 mM IPTG and transferred to 20°C for overnight expression. Cell pellets were harvested by centrifugation at 5000 xg for 20 min at 4°C, then stored at -20°C until purification.

P19 purification. Bacterial pellets were resuspended in cold sonication buffer (2x PBS containing 3% glycerol, 1% triton X-100 and EDTA-free protease inhibitors (Roche), pH 7.5) and lysed by sonication on ice. Following centrifugation at 20,000 xg for 60 min, the supernatant was incubated with cobalt resin (Clontech) for 1 hr at 4°C. The resin was then transferred to a gravity flow column and washed with increasing concentrations of imidazole (2x PBS containing 5 mM, 10 mM and 15 mM imidazole, pH 7.5). A wash step with 2x PBS containing 1 M NaCl was included in-between the steps with 5 mM and 10 mM imidazole, to aid in the removal of non-specifically bound nucleic acids. Sumo-tagged p19 was eluted with 2x PBS containing 250 mM imidazole and concentrated to a smaller volume. The sumo tag was removed from p19 by overnight digestion with sumo protease (added at a 1:40 ratio by mass) at 4°C while dialyzing into 1x PBS. Cleaved sumo and sumo protease, both of which contain his tags, were removed by incubation with cobalt resin (Clontech) for 1 hr at 4°C. The flow-through containing cleaved p19 was collected and buffer-exchanged into 20 mM bis-tris containing 10 mM NaCl, pH 6.5. Anion exchange chromatography (AEX) was performed using a HiTrap Q HP anion exchange column (GE Healthcare Life Sciences) with an increasing salt gradient from 10 mM to 500 mM NaCl. A small peak absorbing strongly at 260 nm separated early, likely containing fugitive nucleic acids. The dominant peak eluting at approximately 200 mM NaCl was collected and concentrated for subsequent size-exclusion chromatography (SEC). Preparatory SEC was performed using a HiLoad 16/600 Superdex 75 pg column (GE Healthcare Life Sciences) in PBS, where p19 eluted mostly as a monomeric peak. Final samples in PBS were sterile-filtered then flash frozen in single-use aliquots, and stored at -80°C.

2. Parameter measurements

Steady-state GFP expression levels. The number of GFP molecules expressed in untreated A431-d2EGFP cells was calculated using AcGFP/EGFP flow cytometer calibration beads (Clontech) on an Accuri C6 cytometer (BD Accuri Cytometers). Measurements were performed during multiple passages, the median value of which was used.

Net internalization rate of EGFR. An EGFR binder engineered on the fibronectin scaffold (clone EI3.4.3 (16)) was labeled with Alexa Fluor 647 following manufacturer's instructions (Life Technologies). The labeled binder was incubated with A431 cells at 20 nM in complete media for 0 – 25 hours in a reverse time course. Incubation was performed either in the presence or absence of excess competitor (unlabeled EI3.4.3) to determine the degree of non-specific uptake, if any. The background-subtracted fluorescence at each time point was fitted to obtain a first-order rate of internalization.

Supplementary References

1. Spangler,J.B., Neil,J.R., Abramovitch,S., Yarden,Y., White,F.M., Lauffenburger,D.A. and Wittrup,K.D. (2010) Combination antibody treatment down-regulates epidermal growth factor receptor by inhibiting endosomal recycling. *Proc. Natl. Acad. Sci.*, **107**, 13252–13257.

2. DeWitt,A.E., Dong,J.Y., Wiley,H.S. and Lauffenburger,D.A. (2001) Quantitative analysis of the EGF receptor autocrine system reveals cryptic regulation of cell response by ligand capture. *J. Cell Sci.*, **114**, 2301–2313.

3. Bartlett,D.W. and Davis,M.E. (2006) Insights into the kinetics of siRNA-mediated gene silencing from live-cell and live-animal bioluminescent imaging. *Nucleic Acids Res.*, **34**, 322–333.

4. Ahlberg,J., Berkenstam,A., Henell,F. and Glaumann,H. (1985) Degradation of short and long lived proteins in isolated rat liver lysosomes. Effects of pH, temperature, and proteolytic inhibitors. *J. Biol. Chem.*, **260**, 5847–5854.

5. Chiu,Y.-L. and Rana,T.M. (2003) siRNA function in RNAi: A chemical modification analysis. *RNA*, **9**, 1034–1048.

6. Bertrand,J.-R., Pottier,M., Vekris,A., Opolon,P., Maksimenko,A. and Malvy,C. (2002) Comparison of antisense oligonucleotides and siRNAs in cell culture and in vivo. *Biochem. Biophys. Res. Commun.*, **296**, 1000–1004.

7. Schwanhäusser,B., Busse,D., Li,N., Dittmar,G., Schuchhardt,J., Wolf,J., Chen,W. and Selbach,M. (2011) Global quantification of mammalian gene expression control. *Nature*, **473**, 337–342.

8. Wittrup,A., Ai,A., Liu,X., Hamar,P., Trifonova,R., Charisse,K., Manoharan,M., Kirchhausen,T. and Lieberman,J. (2015) Visualizing lipid-formulated siRNA release from endosomes and target gene knockdown. *Nat. Biotechnol.*, **33**, 870–876.

9. Darzacq,X., Shav-Tal,Y., de Turris,V., Brody,Y., Shenoy,S.M., Phair,R.D. and Singer,R.H. (2007) In vivo dynamics of RNA polymerase II transcription. *Nat. Struct. Mol. Biol.*, **14**, 796–806.

10. Li,X., Zhao,X., Fang,Y., Jiang,X., Duong,T., Fan,C., Huang,C.-C. and Kain,S.R. (1998) Generation of Destabilized Green Fluorescent Protein as a Transcription Reporter. *J. Biol. Chem.*, **273**, 34970–34975.

11. French,A.R. and Lauffenburger,D.A. (1997) Controlling receptor/ligand trafficking: Effects of cellular and molecular properties on endosomal sorting. *Ann. Biomed. Eng.*, **25**, 690–707.

12. Bryan,A.K., Hecht,V.C., Shen,W., Payer,K., Grover,W.H. and Manalis,S.R. (2013) Measuring single cell mass, volume, and density with dual suspended microchannel resonators. *Lab. Chip*, **14**, 569–576.

13. Choi,K., Park,G.L., Hwang,K.Y., Lee,J.-W. and Ahn,H.J. (2013) Efficient siRNA Delivery into Tumor Cells by p19-YSA Fusion Protein. *Mol. Pharm.*, **10**, 763–773.

14. Schreiber,G. (2002) Kinetic studies of protein–protein interactions. *Curr. Opin. Struct. Biol.*, **12**, 41–47.

15. Koukiekolo,R., Sagan,S.M. and Pezacki,J.P. (2007) Effects of pH and salt concentration on the siRNA binding activity of the RNA silencing suppressor protein p19. *FEBS Lett.*, **581**, 3051–3056.

16. Hackel,B.J., Neil,J.R., White,F.M. and Wittrup,K.D. (2012) Epidermal growth factor receptor downregulation by small heterodimeric binding proteins. *Protein Eng. Des. Sel.*, **25**, 47–57.

17. Rawlings,R.A., Krishnan,V. and Walter,N.G. (2011) Viral RNAi Suppressor Reversibly Binds siRNA to Outcompete Dicer and RISC via Multiple Turnover. *J. Mol. Biol.*, **408**, 262–276.

18. Johnson,B.B. and Heuck,A.P. (2014) Perfringolysin O Structure and Mechanism of Pore Formation as a Paradigm for Cholesterol-Dependent Cytolysins. In Anderluh,G., Gilbert,R. (eds), *MACPF/CDC Proteins - Agents of Defence, Attack and Invasion*, Subcellular Biochemistry. Springer Netherlands, pp. 63–81.

19. Masliah,G., Barraud,P. and Allain,F.H.-T. (2012) RNA recognition by double-stranded RNA binding domains: a matter of shape and sequence. *Cell. Mol. Life Sci.*, **70**, 1875–1895.

20. Yang,N.J., Liu,D.V., Sklaviadis,D., Gui,D.Y., Vander Heiden,M.G. and Wittrup,K.D. (2015) Antibody-Mediated Neutralization of Perfringolysin O for Intracellular Protein Delivery. *Mol. Pharm.*, **12**, 1992–2000.
